# Anti-P antibodies that impair memory perturb hippocampal glutamatergic receptor trafficking, synapse structure and microglia

**DOI:** 10.1186/s10020-025-01339-7

**Published:** 2025-09-26

**Authors:** Nicole Díaz-Valdivia, Mariana Labarca, Claudio Retamal, Sofia Espinoza, Jaime Venegas, Alejandra Catenaccio, Adely de la Peña, Micaela Ricca, Claudia Jara, Daniela Cortés-Díaz, Angela Campos, Francisca Pérez-Molina, Francisca Barake, Bernardita Medel, Cristian Herrera-Cid, Fanny Guzman, Bredford Kerr, Manuel Varas-Godoy, Marcela Bravo-Zehnder, Loreto Massardo, Cheril Tapia-Rojas, Alfonso González

**Affiliations:** 1 Centro de Biología Celular y Biomedicina (CEBICEM), Facultad de Ciencias, Universidad San Sebastián, Av. Del Valle Norte 725, 8580704 Huechuraba, Santiago Chile; 2 Facultad de Ciencias, Universidad San Sebastián, 7510157 Santiago, Chile; 3 Facultad de Medicina, Universidad San Sebastián, 7510157 Santiago, Chile; 4 Departamento de Ciencias Biológicas y Químicas, Facultad de Ciencias, Universidad San Sebastián, 7510157 Santiago, Chile; 5 Centro Científico y Tecnológico de Excelencia, Ciencia & Vida, Fundación Ciencia & Vida, 8580704 Huechuraba, Santiago Chile; 6 Núcleo de Biotecnología Curauma, Pontificia Universidad Católica de Valparaíso, Av. Universidad 330, 2373223 Curauma, Valparaíso Chile; 7 Present address: Centro de Observación y Análisis de Datos en Salud (CADS), Facultad de Medicina y Ciencias de La Salud, Universidad Mayor, 8580745 Huechuraba, Santiago Chile; 8 Present address: Laboratory of Normal and Pathological Homeostasis of the Immune System, INSERM UMR 1163, Image Institute, Paris, France; 9 Present Address: Cilia Group, Department of Biology, University of Copenhagen, Universitetsparken 13, Copenhagen Ø, Denmark

**Keywords:** Neuropsychiatric SLE, Cognitive dysfunction, Anti-ribosomal P antibodies, Neuronal-surface-P-antigen, NSPA, Synaptic plasticity, NMDAR and AMPAR trafficking, PTPMEG, PSD-95

## Abstract

**Background:**

Anti-ribosomal P protein autoantibodies (anti-P) are associated with psychosis and cognitive dysfunction in patients with systemic lupus erythematosus (SLE), yet the underlying mechanisms remain undefined, hindering targeted therapies. Anti-P cross-react with a neuronal surface protein (NSPA), alter glutamatergic synaptic transmission and plasticity in hippocampal slices, and impair spatial memory in a short-term passive transfer mouse model. NSPA knockout mice display spatial memory deficit linked to reduced NMDAR activity and postsynaptic density (PSD) levels, along with an increased membrane-associated tyrosine phosphatase PTPMEG, suggesting disrupted glutamatergic receptor trafficking. Here, we investigated the acute effects of anti-P on receptor cell surface expression and trafficking in cultured hippocampal neurons and their long-term impact on hippocampal components and spatial memory in anti-P( +) immunized mice.

**Methods:**

NMDAR and AMPAR surface expression and NMDAR recycling were assessed in 21-24 DIV primary hippocampal neurons by immunofluorescence and FRAP using SEP-tagged receptors under the effects of rabbit anti-P IgG fractions. In vivo, female C57BL/6 mice were immunized with recombinant P0 ribosomal protein to induce anti-P, followed by lipopolysaccharide (LPS) intraperitoneal administration to breach the blood-brain-barrier (BBB). Spatial memory was evaluated with a water maze memory flexibility test. Hippocampal synaptosomal membranes and PSD-enriched fractions were analyzed by immunoblotting. Neuronal density, microglia and dendritic architecture were evaluated using Cresyl Violet, Iba1 and Golgi staining, respectively.

**Results:**

Anti-P treatment of cultured neurons reduced GluN2A and GluA1 surface levels and impaired SEP-GluN2A and SEP-GluN2B recycling. Anti-P( +) mice showed spatial memory deficits persisting up to 24 days post-LPS, along with hippocampal alterations that include reduced levels of NMDAR, AMPAR, and PSD-95 in PSD fractions; increased membrane-associated PTPMEG; ~ 7% neuronal loss; higher number of microglia with reduced ramifications, and diminished dendritic width and spine density. Notably, increased PTPMEG levels were already detectable by day 10 post-LPS.

**Conclusions:**

Anti-P antibodies acutely impair glutamatergic receptor recycling and surface expression, while their long-term effects lead to sustained memory impairment associated with altered neuronal and microglial architecture, and PTPMEG increased levels preceding PSD protein loss. These findings provide mechanistic insight into anti-P–mediated cognitive dysfunction and may inform therapeutic strategies for neuropsychiatric SLE.

**Supplementary Information:**

The online version contains supplementary material available at 10.1186/s10020-025-01339-7.

## Background

Anti-ribosomal P protein antibodies (Anti-P) in patients with systemic lupus erythematosus (SLE), a chronic autoimmune disease, have been associated with psychosis (Bonfa et al. 1987; Viana et al. 2017; Bonfa and Elkon 1986) and cognitive dysfunction (CD) (Massardo et al. 2015), two neuropsychiatric manifestations that significantly impair patient quality of life (Massardo et al. 2015; Chang et al. 2015) and remain challenging in terms of diagnosis, pathogenesis, and treatment (Govoni and Hanly 2020; Bertsias and Boumpas 2010; Schwartz et al. 2019). Anti-P are present in 15–30% of SLE patients, with prevalence varying by ethnicity and age of disease onset (Viana et al. 2017; Gonzalez and Massardo 2018; Barake et al. 2022). In contrast to the significant advances regarding anti-NMDAR antibodies (DNRabs) involved in lupus-related CD (Zarfeshani et al. 2021), the neuropathogenic mechanisms of anti-P remain poorly understood. Investigating the neuronal components and processes targeted by anti-P in the brain is crucial to better understand these difficult-to-treat neuropsychiatric manifestations of SLE (NPSLE).

Our previous studies have shown that anti-P, when intravenously (i.v.) injected into mice, induce spatial memory impairment, but only when the blood–brain barrier (BBB) is breached by lipopolysaccharide (LPS) (Bravo-Zehnder et al. 2015). In hippocampal CA3-CA1 synapses, anti-P enhance both NMDAR- and AMPAR-mediated transmission, generating a condition that impairs long-term potentiation (LTP) (Segovia-Miranda et al. 2015), the cellular correlate of memory formation (Neves et al. 2008). Anti-P applied to primary cultures or delivered via stereotaxic injection into the brain trigger neuronal apoptosis, likely due to calcium influx-mediated excitotoxicity (Bravo-Zehnder et al. 2015; Matus et al. 2007). Other studies have shown that intraventricular injection of anti-P induces electrophysiological abnormalities and behavioral disturbances (Gaburo et al. 2017), as well as depression-like behavior and olfactory deficits (Katzav et al. 2008, 2007). These findings reflect relatively short-term effects of anti-P. However, their long-term impact on the brain, which may better explain their association with the CD or psychosis in SLE patients (Bonfa et al. 1987; Viana et al. 2017; Massardo et al. 2009), remains unexplored.

The immunodominant P epitope recognized by anti-P lies within an 11-residue C-terminal linear sequence shared by three ribosomal phosphoproteins, P0 (38 kDa), P1 (19 kDa), and P2 (17 kDa), which are components of the large (60S) ribosomal subunit (Elkon et al. 1988; Mahler et al. 2003). Although anti-P have been studied for decades, initially motivated by the report describing an association with psychosis (Viana et al. 2017), their pathogenic mechanisms in the brain remain incompletely understood. For a long time, the role of anti-P in NPSLE was controversial largely due to the absence of a defined neuropathogenic mechanism and the assumption that their targets were strictly intracellular (Viana et al. 2017). Initial studies in human hepatoma cells revealed the presence of a cell surface P antigen (Koren et al. 1992). Much later, we identified a neuronal surface cross-reactive protein, termed NSPA (Matus et al. 2007), which presents a P-epitope at the neuronal cell surface and is expressed in brain regions involved in behavior, emotion, and cognition, including the hippocampus (Segovia-Miranda et al. 2015; Matus et al. 2007). Notably, the acute effects of anti-P on calcium influx and glutamatergic stimulation require NSPA expression, as demonstrated in hippocampal neurons from NSPA-KO mice (Segovia-Miranda et al. 2015).

The characterization of NSPA-knockout mice, either expressing a truncated version or entirely lacking NSPA (both referred to as NSPA-KO) (Segovia-Miranda et al. 2015; Espinoza et al. 2020), provides valuable clues for exploring the long-term neuropathogenic mechanisms of anti-P antibodies. The NSPA-KO mouse phenotype includes alterations in the glutamatergic system (Segovia-Miranda et al. 2015; Espinoza et al. 2020). These mice exhibit spatial memory and LTP impairments associated with reduced NMDAR activity (Segovia-Miranda et al. 2015) and decreased levels of NMDARs at the postsynaptic density (PSD) in the hippocampus, without detectable changes in the AMPAR activity and PSD levels (Segovia-Miranda et al. 2015; Espinoza et al. 2020). PSD-95, a core scaffolding protein of the PSD that interacts with NMDARs and AMPARs, stabilizing them at the synapse (Shahar et al. 2024), also remains unchanged in the NSPA-KO mice (Espinoza et al. 2020). However, a decreased phosphorylation of GluN2B-Tyr1472 (Espinoza et al. 2020), known to promote endocytosis of GluN2B-containing NMDARs (Won et al. 2019), may explain the lower levels of NMDAR at the PSD (Espinoza et al. 2020).

The best-characterized tyrosine phosphorylation system in neurons involves Src and Fyn kinases, which phosphorylate and stabilize NMDARs and AMPARs at the cell surface, whereas the tyrosine phosphatase STEP61 promotes their endocytosis (Won et al. 2017; Won and Roche 2021; Lombroso et al. 2016). Interestingly, NSPA-KO mice show normal levels of Fyn and STEP61 compared to WT controls but display increased levels of the megakaryocyte protein tyrosine phosphatase (PTPMEG) in hippocampal synaptosomal membranes (Espinoza et al. 2020). PTPMEG, also known as protein tyrosine phosphatase non-receptor type-4 (PTPN4), is highly expressed in the brain (Szczaluba et al. 2018; Gu and Majerus 1996), contributes to neuronal function (Barake et al. 2022; Szczaluba et al. 2018; Williamson et al. 2015; Kohda et al. 2013a; Kina et al. 2007), and its subcellular distribution includes the plasma membrane and dendritic spines in hippocampal neurons (Szczaluba et al. 2018). Collectively, the evidence supports a role for NSPA as an E3-ubiquitin ligase, with PTPMEG as one of its substrates involved in the trafficking and stabilization of NMDAR at the PSD (Barake et al. 2022; Espinoza et al. 2020).

To advance the understanding of anti-P pathogenicity, it is essential to investigate whether the previously described acute effects of anti-P leading to LTP impairment (Segovia-Miranda et al. 2015) involve alterations in the surface expression and trafficking of ionotropic glutamatergic receptors implicated in cognition and memory (Citri and Malenka 2008). The trafficking of NMDARs and AMPARs through endocytic and recycling pathways, as well as their lateral diffusion at the plasma membrane, contributes to synaptic plasticity and memory formation (Beattie et al. 2000; Groc and Choquet 2020; Storey et al. 2025; Yang et al. 2022). LTP, in particular, depends on increased AMPAR levels at the synapse (Groc and Choquet 2020; Storey et al. 2025; Yang et al. 2022; Granger et al. 2013).

In parallel, it is also crucial to define whether long-term effects of anti-P include memory impairments and to what extent they recapitulate the synaptic alterations observed in the absence of NSPA expression (Espinoza et al. 2020). A well-characterized experimental model of antibody-induced CD in NPSLE, based on a subset of anti-double-stranded DNA antibodies known as DNRabs, which cross-react with NMDARs, highlights neuronal death, microglia activation, and neuronal structural features as additional aspects to investigate regarding the pathogenicity of autoantibodies associated with CD (Nestor et al. 2018; Carroll et al. 2024).

Here, using primary hippocampal neurons, we first show that the acute effects of anti-P include reduced surface levels of NMDARs and AMPARs, along with retarded NMDAR recycling in synaptic spines. In vivo, we demonstrate that the long-term effects of anti-P, assessed in P0-immunized mice, include spatial memory impairment at both 10 and 24 days after LPS-mediated BBB breaching. This memory impairment is associated with increased PTPMEG levels at both time points, while other synaptic components display time-dependent variations. Notably, by 24 days post-BBB breaching, the PSD levels of not only NMDARs but also AMPARs and PSD-95, are significantly reduced, indicating a substantial compromise of synaptic structure and function. In addition, hippocampal tissue analysis revealed approximately 7% neuronal loss, increased microglia presence with reduced ramifications, and decreased synaptic spine density. These findings provide mechanistic insight into how anti-P antibodies may alter synaptic structure and function. Our mouse model highlights the long-term effects of anti-P antibodies and underscores the need for further studies to elucidate how these alterations might be mechanistically interconnected, potentially complementing the effects of DNRabs (Nestor et al. 2018; Carroll et al. 2024), and translated into therapeutic strategies for cognitive dysfunction in NPSLE.

## Methods

### Animals

All animal experiments were approved by the Ethical Committee of Universidad San Sebastián (07–24 and 09–22 codes). Female C57BL/6 mice were maintained in standard cages under constant temperature (21ºC) and in a 12:12 light–dark cycle, with free access to tap water and food, and were used between 10–12 weeks of age.

### Antibodies

Purchased antibodies used for immunoblots include primary monoclonal mouse antibodies from Biolegend or Developmental Studies Hybridoma Bank (DSHB) against GluN1 (N308/48) (Cat#75–272, RRID:AB_11000180, 1:1000), GluN2A (N327/95) (Cat#75–288, RRID:AB_2315842, 1:500), GluN2B (N59/36) (Cat#75–101, RRID:AB_2232584, 1:1000), GluA1 (N355/1) (Cat#75–327,RRID:AB_2315840, 1:1000), GluA2 (L21/32) (Cat#75–002, RRID:AB_2232661, 1:1000), and PSD-95 (K28/43) (Cat#75–028, RRID:AB_2292909, 1:10,000), and from other sources against β-actin **(**Cell Signaling Technology Cat#3700**,** RRID:AB_2223210, 1:1000), STEP (clone 23E5) (Millipore Cat#05–730, RRID:AB_11212456, 1:1000), phospho-GluN2B (Tyr1472) (Cell Signaling Technology Cat#4208, RRID:AB_1549657, 1:1000), anti-Synapsin I (Abcam Cat#ab8, RRID:AB_2200097, 1:10,000). For immunofluorescence analyses, monoclonal antibodies against GluA1 (N355/1) and GluN2B (N59/36) extracellular domains were used at a 1:100 dilution. Polyclonal rabbit antibodies against PTPMEG were produced in house by immunizing with the peptide EEGFVKPLTTSTNK-COOH corresponding to the last 14 amino acids of NP_002821.1, previously probed to generate these antibodies (Gu and Majerus 1996). The peptide was synthesized following described methods (Santana et al. 2013) and was coupled to mollusk *Megathura crenulata* hemocyanin (Sigma-Aldrich, #H8283), under published protocols (Matus et al. 2007; Salazar and Gonzalez 2002; Cancino et al. 2007). Secondary antibodies include horseradish peroxidase (HRP)-conjugated-antibodies (Rockland) for immunoblot, alkaline phosphatase (AP)-conjugated-antibodies for ELISA and Alexa-conjugated antibodies (Invitrogen) for immunofluorescence.

### Plasmids

pCI-SEP-GluN2A (Addgene plasmid #23997 http://n2t.net/addgene:23997; RRID:Addgene_23997), pCI-SEP-GluN2B (Addgene plasmid #23998; http://n2t.net/addgene:23998; RRID:Addgene_23998) were obtained from Addgene.

### Hippocampal primary cultures

Pregnant Sprague–Dawley rats were deeply anesthetized with CO_2_, decapitated and E18 embryos (6–12 per pregnant rat) quickly removed and decapitated. Embryos were dissected to obtain brain hippocampi in ice cold Hanks'Balanced Salt Solution pH 7.4 (HBSS) (Thermo Cat#14175095). Tissue was digested with 0.25% trypsin (Thermo Cat#25200056) for 15 min in HBSS at 37 °C, trypsin was removed and tissue was mechanically disrupted by serial passage through fire-polished glass Pasteur pipettes of decreasing diameters. Disaggregated cells were resuspended in adhesion media (DMEM, 10% horse serum (Hyclone Cat#SH30074.03), 100U/ml Penicillin/Streptomycin) counted and plated in poly-D-Lysine (Thermo Cat#A3890401) coated 35 mm glass-bottom dishes (700.000 cells) for live cell imaging and glass coverslips (10.000 cells) for immunofluorescence. Cells were allowed to adhere for 3 h and then media was changed to growth medium (Neurobasal (Thermo Cat#21103049) supplemented with 2% B27 (Thermo, Cat#17504044), 1% Glutamax (Thermo Cat#35050061) and 100U/ml Penicillin/Streptomycin). Cultures were not treated with cytosine β-D-arabinofuranoside (Ara-C) to preserve the presence of glial cells as described (Pozzi et al. 2017). Neurons were maintained at 37ºC in 5% CO2 for 21–24 days with half of its growth media replaced twice a week to keep endogenously secreted neurotrophins.

### Intracellular calcium imaging

Primary hippocampal cultures plated in 12 mm glass coverslips were loaded with the ratiometric calcium probe Fura-2 AM (1μM) in culture medium for 1 h and then mounted in a live imaging chamber with filter sterilized Tyrode buffer (NaCl 119 mM, KCl 5 mM, CaCl_2_ 2.5 mM, MgCl_2_ 2.5 mM, HEPES 25 mM, pH 7.3, Glucose 30 mM) at 37 °C. Imaging was performed using a Leica DMI6000b microscope with LasAF software and a 63 × glycerol immersion lens. Excitation was set at 340 and 380 nm whereas emission was collected at 550 nm. Calcium was quantified in ROIs spanning neuron somas by calculating the 340/380 emission ratio in images taken at each 10 seg, as described (Bravo-Zehnder et al. 2015; Segovia-Miranda et al. 2015; Oakes et al. 1988).

### Neuron immunofluorescence

Primary hippocampal cultures of 21–24 DIV maintained in 12 mm coverslips and treated as indicated were washed in warm PBS-CM (PBS, CaCl2 1 mM, MgCl2 10 mM) and fixed at room temperature (RT) with 4% PFA 4% Sucrose in PBS for 15 min. Cells were incubated with anti-GluA1 or anti-GluN2A (DSHB) in PBS 30 min at 37ºC. Coverslips were washed and incubated with Alexa 568-coupled secondary anti-mouse antibody for 30 min at 37ºC, washed and mounted in Fluoromount. Images were acquired in a Leica TCS SP8 spectral confocal microscope (× 63 oil immersion objective, 1.4 N.A.) running the LASX Leica software.

### Transfection and FRAP assays

Primary hippocampal cultures plated in 35 mm glass-bottom dishes were transfected at DIV-8 using a Lipofectamine 2000 to DNA ratio of 2:1 in Optimem, following the manufacturer’s instructions. Superecliptic pHluorin pCI-SEP-GluN2B was used at 1 µg/dish and pCI-SEP-GluN2A at 0.3 µg/dish. Transfection media was replaced after 1 h with medium previously saved from the same cells, to allow recovery in a neurotrophin-rich environment and were then maintained as mentioned before. At 21–24 DIV cells were placed in a 37ºC-controlled temperature recording chamber filled with Tyrode buffer (137 mM NaCl, 5 mM KCl, 2 mM CaCl2, 1 mM MgCl2, 20 mM Glucose, 10 mM Hepes, pH 7.4). Transfected neurons were visualized in a Leica TCS SP8 spectral confocal microscope (× 63 oil immersion objective, 1.4 N.A.) running the LASX Leica software. Regions of interest (ROIs) of 50—100 μm were defined at dendrites and photobleached with 3 × 5 pulses at 488 nm laser using FRAP booster and 100% laser-power, then recorded every 30 s with autofocus control. Videos were analyzed offline and the recovery of fluorescence at dendritic spines, defined by small circular ROIs, was measured. ROIS were randomly selected at places with easily detected matured spines (more than 2 µm long). Total fluorescence intensity was quantified.

### Expression and purification of recombinant P0

P0 was cloned into pGEX-4 T-3 plasmid by PCR from a human kidney cDNA library (BD Biosciences Clonetech) using forward primer 5’-GGAATTCGATGCCCAGGGAAGACAG-3´ and reverse primer 5’-CCGCTCGAGTTAGTCAAAGAGACCAAATCC-3´. A truncated version of P0 (trP0), lacking the P epitope, was cloned from pGEX4T-3-P0 using the same forward primer used for building GST-P0 and reverse primer 5’-CCGCTCGAGAATCAACTCCTCCGACTCTTCCT-3´, using EcoI and XhoI restriction enzyme sites coded within primers, according to standard procedures. Recombinant P0 and trP0 were isolated from *E. Coli* strain DH-5a transformed with these plasmids. The recombinant proteins were induced with 1 mM IPTG (US Biological, Cat#I8500) for 4 h at 25 °C and purified by glutathione-Sepharose affinity chromatography (100uL per 100 mL bacterial culture) (Cytiva, Cat#17061801) according to the manufacturer's instructions. Recombinant proteins were released from glutathione sepharose resin by thrombin cleavage (2U per 100 mL bacterial culture) (Sigma-Aldrich, Cat#T1063). The amount of recombinant protein was estimated in acrylamide gel electrophoresis and Coomassie blue staining.

### Immunization and opening of the blood–brain barrier

C57BL6 female mice, 10–12 week-old, were immunized with three intraperitoneal (i.p.) injections of P0 (100 µg) recombinant protein or its suspension medium (PBS), with 2 weeks intervals, 6 mice per group, according to the scheme in Fig. 2A. The age range depended on the purpose of the experiment. If the time period for the memory test was 10 days after BBB opening, the initial age was 12 weeks, whereas for the experiments at 24 days post BBB opening, the initial age was 10 weeks, thus allowing to evaluate the effect of memory and hippocampal protein components at the same age (20 weeks).The firs injection was made by mixing P0 with one volume of complete Freund’s co-adjuvant, or PBS-Freund’s co-adjuvant in control animals, in a maximum volume of 100-200uL per mouse. The two boosters with P0 protein or PBS were made in incomplete Freund’s co-adjuvant (Thermo Scientific) using the same volume of the first injection. Serum was obtained 24 h before each immunization to test anti-P antibodies in mice by P11-peptide ELISA. The BBB was breached by 2 i.p. injections of 5 mg/kg of LPS (Sigma-Aldrich, Cat#L3129) dissolved in DMEM, 48 h apart. The behavioral tests were performed at 10 or 24 days after the last LPS injection.

### Enzyme-linked immunosorbent test (ELISA)

The levels of anti-P11 antibodies (epitope recognized by anti-P antibodies) in serum were determined by ELISA assays. Briefly, 96-well ELISA plates were coated with P11 peptide (KSDEDMGFGLFD-COOH, 15 ug/mL, Eurogentec) overnight at 4 °C and then were blocked in PBS BSA 0.1%. A 1/50 dilution of each serum was added, incubated for 1 h and washed 4 times in PBS 0.1% Tween-20. Secondary anti-mouse antibody coupled to alkaline phosphatase was used at 1:1000 dilution. Alkaline phosphatase activity was detected with its substrate (Merck, Cat#ES009) dissolved in Buffer Na_2_CO_3_ 50 mM, MgCl_2_ 1 mM, allowed to develop for 1 h to then measure absorbance at 405 nm. Absorbances over 3–tenfold the median were considered high titers.

### Memory flexibility test

The behavioral memory flexibility test (Bravo-Zehnder et al. 2000), an adaptation of the Morris water maze protocol, was performed 10 or 24 days post-injection with LPS. The learning criterion was to find 3 consecutive times a hidden platform in the water, which changes quadrant every day, with an average arrival time ≤ 20 s, without exceeding 15 attempts per day. These tests were performed during 4 consecutive days.

### Subcellular fractionation of mice hippocampi and immunoblot

After the memory tests, mice were euthanized, the hippocampi of both hemispheres removed and snap frozen in liquid nitrogen. For protein analysis, hippocampi were homogenized in sucrose suffer (320 mM sucrose, 3 mM EDTA, 10 mM Hepes) supplemented with protease and phosphatase inhibitor Mini Tablets (Pierce #A32959, Thermo Fisher Scientific) using a Potter homogenizer. The homogenates (H) were centrifuged twice at 1000 g for 10 min at 4 °C and the pellet P1 was eliminated. Supernatant 1 (SN1) was centrifuged at 12,000 g for 20 min at 4 °C generating the pellet P2 and supernatant 2 (SN2) that was stored at −80ºC. P2 was resuspended in cold Triton Buffer (0.5% Triton X-100 in 10 mM Tris–HCl pH 7.2, 100 mM NaCl), gently stirred for 15 min at 4 °C, and centrifuged at 12,000 g for 20 min at 4ºC, generating the supernatant Triton-soluble fraction containing non-PSD membranes (Zamzow et al. 2016). The resulting pellet was resuspended in SDS Buffer (10 mM Tris–HCl pH 7.5, 150 mM NaCl, 1% SDS, 1 mM DTT, 1% Deoxycholate, 1% Triton X-100), gently stirred for 1 h at 4 °C, then centrifuged at 10,000 g per minute 15 min at 4 °C, obtaining the supernatant (TxP) that contains PSD (Zamzow et al. 2016). Protein concentrations were determined using the BCA assay (Pierce, Thermo Fisher Scientific). Protein fractions were separated by SDS-PAGE on discontinuous 12 and 7.5% acrylamide gels, transferred to PVDF membranes, and then subjected to standard immunoblot procedures.

### Brain histochemistry and immunofluorescence

For brain perfusion and post-fixation**,** mice were anesthetized by intraperitoneal administration of 200 μL of a ketamine/xylazine solution (100 mg/kg, 5 mg/kg, respectively) and transcardiac perfusion was performed using phosphate-buffered saline (PBS 1x) as a washing solution, followed by 4% paraformaldehyde (PFA, Merck #158127) as fixative. After perfusion, brains were extracted and post-fixed in 4% PFA overnight at 4°C. The tissues were serially transferred to sucrose solutions in PBS starting with a 10% concentration for 3 h, then a 20% solution for an additional 3 h, and finally a 30% sucrose solution overnight at 4°C. For tissue sectioning, the brains were mounted with Tissue-Tek O.C.T. Compound (Sakura, #4583) and sectioned on a cryostat (Leica CM1860 UV) in coronal slices of 25 μm thickness from anterior to posterior. All hippocampus sections were collected in a plate with PBS 1 × and kept at 4º until use. The tissues were mounted into positively charged slides (Color Code Plus, PR Lab, #PC2-302–16) for use. Cresyl violet staining was performed after washing the slides briefly in tap water to remove residual salt. The slides were then submerged in Cresyl violet solution for 5 min (0.3% Cresyl Violet (Sigma-Aldrich, #C3886), 0.1% Acetic Acid (Merck, #100063) in H_2_O), washed in tap water to remove excess stain and then immersed 3 min in 95% Ethanol and 2 min in 100% Ethanol. Finally, slides were submerged 2 times in Xylol, 5 min each, and mounted in Eukitt Quick-hardening mounting medium (Sigma-Aldrich, #03989). Images were obtained in a stereological microscope, Olympus BX51, at 4x (whole brain) and 20x (CA1 region). A total of eleven sections per group (n = 3) were analyzed: four sections from each of two animals, and three sections from the third animal. For each mouse, three sections were randomly selected from the central hippocampus region (corresponding to plate 49 of the Paxinos and Franklin atlas). The distance between adjacent sections was 100 μm. Neuronal counts were performed within a defined ROI of 200 × 200 μm, and results were normalized to 100 μm^2^ of hippocampal volume, to allow comparison across animals, considering potential volume differences. Nuclei were counted in a 200 × 200 µm box and normalized to 100 µm^2^. Nuclei in the specific area were counted using Image J software. For brain immunofluorescence**,** the slides were hydrated in PBS 1x, incubated in Blocking/Permeabilization Solution (Triton X-100 0.5%, BSA 5% in PBS 1x) for 1 h at room temperature and then incubate at 4ºC overnight with primary rabbit anti-IbaI (Cell Signaling, #17198) antibodies diluted 1:300 in the same solution. After three washes in PBS 1x, 10 min each, slices were incubated with secondary anti-rabbit Alexa 594 (Invitrogen, #A-21207), 1:500 dilution, for 2 h at room temperature, washed again threefold for 10 min each in 1 × PBS and covered in Fluoromount-G (Invitrogen, #00–4958-02) with Hoechst 33342 (Invitrogen, #I35103C).

### Microglia analysis

CA1, CA3 and GD images were obtained in a stereological microscope (Olympus BX51) at 20 × and Iba1 positive cells were quantified in each hippocampal region without considering their morphology and normalized to 100 µm^2^. For analyzing the morphology of microglial cells, 5–10 captures of Iba1 cells were taken at the stratum radiatum of CA1 and CA3 regions, using a Leica TCS SP8 confocal microscope, 63 × oil immersion objective, and Z-stacks slices of 300 nm with 1024 × 1024 pixels resolution. Images were processed with ImageJ software (NIH) to obtain maximal projections, including deconvolution with Huygens Essential software in selected samples, as indicated. Microglia morphology was scored according to their branching using the following criteria: Score 0 = 5 or more thin processes with ramifications; Score 1 = 4–2 processes with or without ramifications; Score 2 = 1 process with or without ramifications; Score 3 = without processes. The distribution frequency of each score was analyzed using a contingency table graphically represented in GraphPad Prism 10.

### Golgi staining

Brains were extracted and immersed in impregnation solution A (potassium dichromate and mercuric chloride) and B (potassium chromate) and kept at room temperature in the dark for 14 days, with the A + B solution replacement after 24 h, following instructions for the FD Rapid GolgiStain™ Kit (FD NeuroTech, #PK401). Tissues were transferred to solution C (silver nitrate) and stored at 4 °C for 48 h, replacing the solution after 24 h. Tissues were sectioned at 100 µm using a cryostat (Leica CM1860 UV) and mounted on glass slides. Sections were incubated with solution D + E for 10 min and rinsed twice in double-distilled water for 4 min each. For dehydration, the samples were treated with 50%, 75%, and 95% ethanol for 4 min each, followed by 4 washes in absolute ethanol 4 min each. The tissues were cleared in xylene 3 times, 4 min each. Finally, the samples were mounted using the Eukitt mounting medium. Images were acquired from three different slices, each from three animals per group in the stratum radiatum of CA1 region, using the 60 × objective of a ZeissImager.M2 Stereologic microscope (z = 0.5 µm). Images were randomly chosen for the central hippocampus (plate 49 from Paxinos Atlas). Image J software was used to analyze the process number and spine density in the maximal projection image. Twenty dendrites randomly selected from each animal (*n* = 3) were analyzed only when measuring 50–100 µm, considering that the secondary and tertiary dendrites of the CA1 of the hippocampus are in this range (Spruston 2008; Megias et al. 2001).

### Statistical analysis

Results are shown as mean ± standard deviation (SD) and proportions as percentages. As indicated in the corresponding figure legends, statistical analysis was performed using GraphPad Prism version 10 for MacOS. An unpaired t-test was performed to compare two experimental groups, and one-way ANOVA followed by Tukey’s multiple comparisons test. Chi-square (χ^2^) test was applied for frequency analyses. *P*-value < 0.05 was considered statistically significant.

## Results

### Anti-P decrease NMDAR and AMPAR cell surface levels and inhibit NMDAR recycling in primary hippocampal neurons

**Fig. 1:**
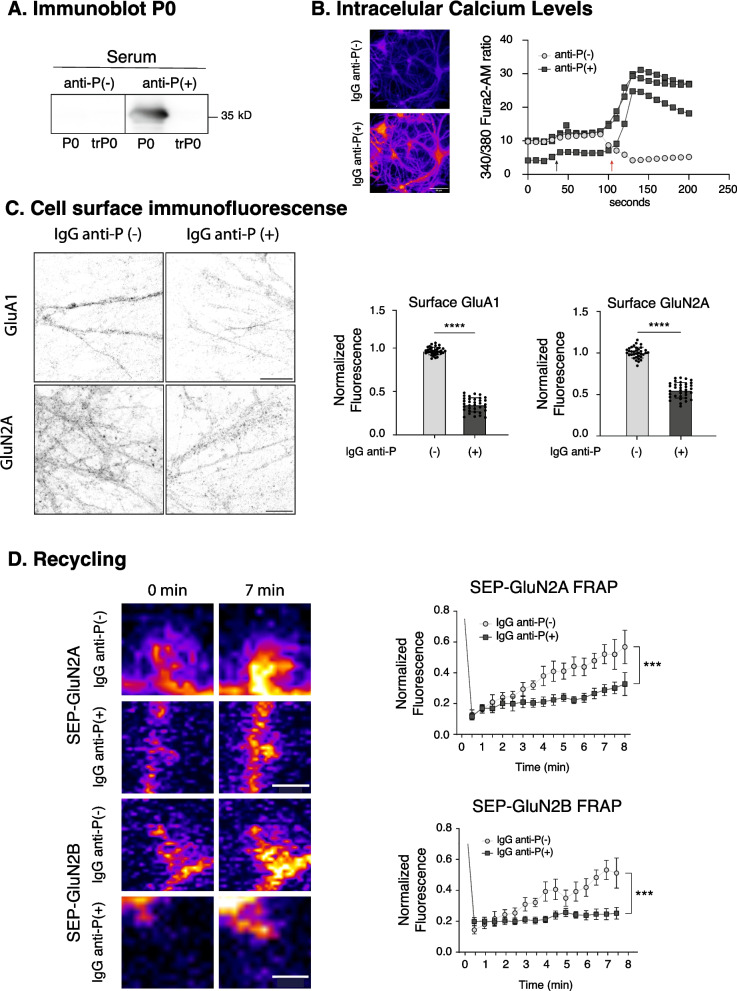
Anti-P decrease NMDAR and AMPAR at the cell surface and impair NMDAR trafficking.** A** Immunoblot showing that rabbit anti-P(+) serum binds to recombinant P0 but not to its truncated version (trP0) lacking the P11-epitope, while the preimmune anti-P(-) serum does not detect any protein. **B-D** Hippocampal neurons (21-24DIV) were incubated with rabbit anti-P(+) of anti-P(-) sera, or the corresponding IgG fractions (10 μg/ml), as indicated below, and then evaluated for the following parameters: **B** Intracellular calcium levels assessed with Fura-2 AM. Representative images of different neurons show that anti-P(+) serum addition (red arrow) elicited rapid calcium increase, contrasting with anti-P(-) serum addition (black arrow); **C** Indirect immunofluorescence of AMPAR-GluA1 and NMDAR-GluN2A subunits after 60 min incubation with either anti-P(+) or anti-P(-) IgG fractions (Bar: 10 μm). Graph shows normalized total fluorescence along neurite ROIs. Anti-P(+) IgG decreased total fluorescence intensity of both GluA1 (~ 60%) and GluN2B (~ 50%), compared to control anti-P(-) IgG. (*n* = 3 per group, analyzing 35 ROIs, mean ± SD; ****p < 0.0001, unpaired t-test). **D** Fluorescence recovery after photobleaching (FRAP) of SEP-GluN2A and SEP-GluN2B. Neuron photobleaching was performed along 100 μm long ROIs, after 15 min of preincubation with anti-P(+) or anti-P(-) IgGs. Fluorescence recovery was analyzed at individual dendritic spines, shown by representative images, every 30 seg. (Bar: 1 μm μm). Graph represents normalized fluorescence over time, showing decreased recovery under anti-P(+) IgG treatment, indicating recycling impairment. (*n* = 3 for SEP-GluN2A and n = 4 for SEP-GluN2B; mean ± SD of at least 30 ROIs in each experiment, ***p < 0.001, Two-way ANOVA, comparison of both curves)

Anti-P antibodies have previously been shown to activate NMDAR and AMPAR resulting in impaired induction of LTP (Segovia-Miranda et al. 2015). Since LTP induction involves the trafficking of these receptors through endocytosis, recycling, and diffusion pathways, leading to higher AMPAR levels at the synaptic region (Groc and Choquet 2020; Yang et al. 2022; Dupuis et al. 2023), we asked whether anti-P affect the cell surface expression of these receptors. To address this, we used previously characterized anti-P obtained from rabbits immunized with an 11-mer synthetic peptide containing the P epitope (Bravo-Zehnder et al. 2015). IgG isolated from this anti-P(+) serum recognizes the recombinant ribosomal P0 but not its truncated version (trP0), which lacks the last 11 C-terminal residues containing the P epitope (Fig. 1A). In contrast, IgG from the pre-immune anti-P(-) serum used as control did not recognize either protein (Fig. 1A). These IgG fractions were applied to primary hippocampal neurons cultured for 21–24 days in the presence of glial cells (Supplementary Fig. 1 A), a condition known to support healthy neurons with mature dendritic spines (Pozzi et al. 2017; Kaech and Banker 2006).

Given that anti-P has been shown to induce calcium influx in neurons (Matus et al. 2007), we first verified their biological activity by assessing intracellular calcium responses using the calcium reporter Fura-2 AM (Bravo-Zehnder et al. 2015; Segovia-Miranda et al. 2015; Oakes et al. 1988). This step also ensured the quality of the neuronal cultures. Anti-P triggered a rapid increase in intracellular calcium levels within seconds (Fig. 1B), confirming antibody activity and neuronal viability. Primary neurons treated with anti-P(+) IgG for 60 min exhibited decreased surface levels of GluA1-AMPAR and GluN2A-NMDAR, as revealed by immunofluorescence (Fig. 1C), while total receptor levels and synaptic spines remained unchanged (Supplementary Figs. 1B and 3), suggesting anti-P-induced trafficking alterations.

To assess receptor recycling, we performed FRAP (fluorescent recovery after photobleaching) experiments using SEP-tagged GluN2A or GluN2B subunits. SEP is a pH-sensitive variant of GFP protein that fluoresces at the neutral pH of the extracellular space but not in the acidic environment of endocytic compartments (Petrini et al. 2009; Kopec et al. 2006). We pretreated hippocampal neurons with anti-P for 15 min prior to photobleaching. To examine receptor reinsertion into the plasma membrane from intraspinal endocytic compartments, while minimizing the contribution of lateral diffusion from adjacent dendritic shaft membranes or endocytic pools, we bleached large dendritic regions (50–100 µm) and assessed FRAP within individual spines at early time points. Analysis on circular ROIs at individual dendritic spines within the first 5 min post-bleaching revealed a clear reduction in SEP-GluN2A and -GluN2B recycling in neurons treated with anti-P (Fig. 1D). To determine whether the observed effect might reflect compromised neuronal health due to the extensive bleached regions, we extended the FRAP analysis to longer time frames and compared the recovery dynamics in anti-P treated neurons with those in untreated controls. Both groups exhibited exponential recovery kinetics, with a rapid initial phase of about 5–7 min followed by a slow phase that reaches a plateau over 20–50 min, similarly across treated and untreated neurons (Supplementary Fig. 2). These dynamics reproduce the typical fluorescence recovery expected for SEP-tagged synaptic receptor constructs (Tatavarty et al. 2013) and indicate that the neurons remained functionally healthy, ruling out a general impairment in receptor trafficking. However, in agreement with the results shown in Fig. 1D, these longer recordings also revealed a delayed fast-recovery component within the first 5 min in anti-P-treated cells (Supplementary Fig. 2). These findings support the conclusion that the acute effects of anti-P impair NMDAR recycling.

### Long-term effects of anti-P lead to memory impairment associated with alterations in the levels of synaptic components

**Fig. 2:**
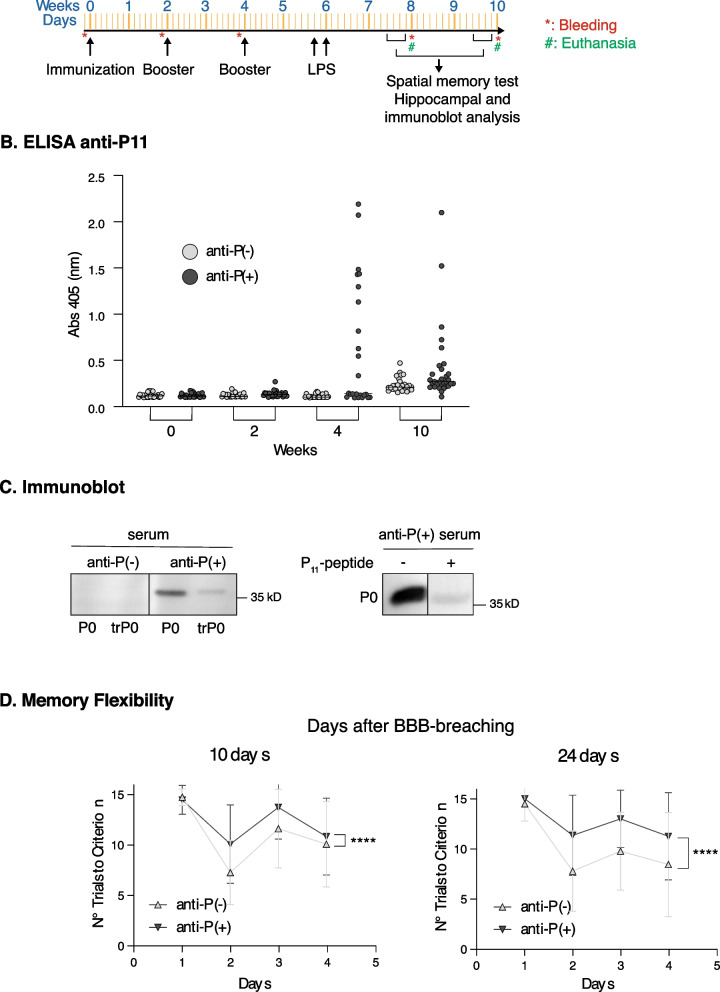
Impaired spatial memory after 10- and 24-days post-BBB breaching in anti-P(+) mice**. A** Scheme with the timing of P0 immunization, LPS treatments, spatial memory tests and immunoblot analysis of hippocampal fractions. **B** ELISA analysis against the synthetic P11-epitope peptide showing increased titer of anti-P antibodies after 4 and 10 weeks post immunization. **C** Immunoblots against recombinant P0 and trP0 lacking the P-epitope and competence with synthetic-P11-peptide. Serum from P0 immunized mice, anti-P(+), but not its corresponding preimmune serum, anti-P(-), binds in immunoblot the P0 protein and not the trP0 protein, and this binding is inhibited with competence with 150 µg/mL of synthetic P11-peptide. **D** Memory flexibility test performed at 10 and 24 days after LPS. anti-P(+) mice shows higher number of trials to meet criterion compared with control anti-P(-) mice indicating impaired memory (mean ± SD; n = 12 per group; ****P < 0.0001, Two-way ANOVA, comparison of both curves)

To evaluate the long-term effects of anti-P on spatial memory and hippocampal synaptic components, we adapted a previously described immunization protocol using recombinant P0, which induces antibodies that recognize a P epitope-containing peptide in ELISA assays (Ben-Ami Shor et al. 2014). (The P0 preparation is shown in Supplementary Fig. 4A-B). We immunized C57BL/6 female mice with recombinant P0 (100 µg) and used co-adjuvant alone for control animals, following the schedule shown in Fig. 2A. ELISA analysis with a synthetic P-peptide confirmed high titers of anti-P two weeks after the second booster, which persisted in some animals for up to eight weeks (Fig. 2A and B). Immunization with lower amounts of P0 (60 µg) did not generate detectable anti-P antibodies (Supplementary Fig. 4 C). Anti-P(+) sera from P0 immunized mice, but not from anti-P(-) control mice, recognized full-length P0 and not the truncated P0 (trP0) lacking the P epitope in immunoblots (Fig. 2C). Moreover, pre-incubation with the synthetic P-peptide blocked this binding, confirming the specificity of the response (Fig. 2C). These results indicate that the sera from P0-immunized mice contain antibodies that specifically target the P-epitope.

Intraperitoneal injection of LPS enables circulating antibodies to access the hippocampus, where they remain detectable for approximately two weeks, as shown for the DNRab mice model (Chang et al. 2015; Nestor et al. 2018; Kowal et al. 2006). After LPS treatment, DNRab antibodies have been shown to impair performance in hippocampus-dependent memory tasks without affecting fear conditioning, which depends on amygdala function (Kowal et al. 2004; Huerta et al. 2006). Our previous studies demonstrated that intravenously injected anti-P also reach the hippocampus following LPS-induced BBB disruption, leading to memory impairment within 2–6 days post-LPS (Bravo-Zehnder et al. 2015). In those experiments, we assessed spatial memory using a spatial memory flexibility test (Bravo-Zehnder et al. 2015), a modified version of the Morris water maze (Chen et al. 2000), conducted only within a narrow window starting two days after BBB-breaching and lasting for four days (Bravo-Zehnder et al. 2015), thus leaving open the question of whether anti-P can impair memory at later time points. To address this question, we now immunized mice with P0 (Fig. 2A and B), then administered two i.p injections of LPS 48 h apart to breach the BBB, and evaluated spatial memory at either 10 or 24 days after the second LPS injection, using the same memory flexibility test previously shown to detect anti-P-induced deficits (Bravo-Zehnder et al. 2015). At both time points, anti-P(+) mice performed worse than anti-P(-) controls, indicating a sustained impairment of spatial memory (Fig. 2D).

This experimental setting allowed us to assess whether the effects of anti-P include alterations previously described in NSPA-KO mice, such as changes in NMDAR levels at PSD, and PTPMEG and GluN2B-pTyr1472 levels in synaptosomal membrane fractions (Espinoza et al. 2020). One day after completing the memory flexibility test, we euthanized the animals, extracted the hippocampi, and performed immunoblot analysis on hippocampal synaptosomal total membranes (P2 fraction) and a Triton X-100 insoluble fraction (TxP) enriched in PSD components (Zamzow et al. 2016). Characterization of these fractions revealed that synapsin-1, a presynaptic marker, was nearly undetectable, while the PSD-95 post-synaptic maker was enriched in the TxP fraction compared to the Triton X-100 soluble fraction, confirming PSD enrichment (Zamzow et al. 2016) (Supplementary Fig. 5).

**Fig. 3:**
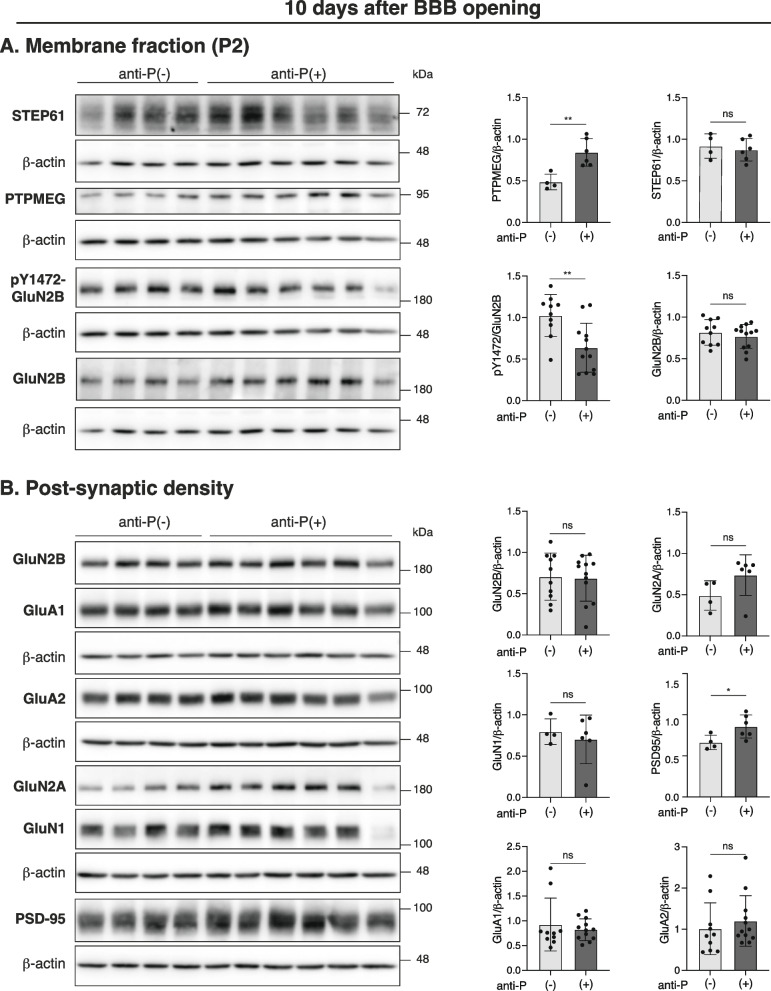
Anti-P effects of synaptosomal membrane and post-synaptic-density (PSD) proteins after 10 days of BBB-breaching with LPSImmunoblot analysis of the indicated proteins and the corresponding quantification graphs normalized against beta-actin. A. Total synaptosomal membrane fractions (P2) show increased levels of PTPMEG (n=4 anti-P(-) group and n=6 anti-P(+) group; mean ± SD; **P < 0.01, unpaired t-test) and decreased GluN2B-Tyr1472 phosphorylation (pY1472) (n=10 anti-P(-) group and n=12 anti-P(+) group; mean ± SD; **P < 0.01, unpaired t-test), while STEP61 shows no differences among the two groups. B. Post-synaptic density (PSD) fractions. NMDAR (GluN2B, GluN2A and GluN1) and AMPAR (GluA1 and GluA2) subunits show no differences, while PSD-95 levels increased (mean ± SD; n= 4 or 12 Anti-P(-) & n= 6 or 12 Anti-P(+) groups; *P < 0.05, unpaired t-test). All proteins were normalized to their corresponding beta-actin bands. pY1472-GluN2B was additionally normalized against GluN2B total (previously normalized for beta-actin)

Ten days after BBB-breaching, we observed increased levels of PTPMEG and reduced phosphorylation of GluN2B at Tyr1472 (pTyr1472-GluN2B) in the synaptosomal membranes of anti-P(+) mice compared to anti-P(-) mice, while STEP61 levels remained similar between groups (Fig. 3A). At this time point, the PSD fractions from anti-P(+) and anti-P(-) mice showed similar levels of NMDAR and AMPAR subunits (Fig. 3B). However, PSD-95 levels were elevated in the PSD fraction of anti-P(+) mice (Fig. 3B).

**Fig. 4:**
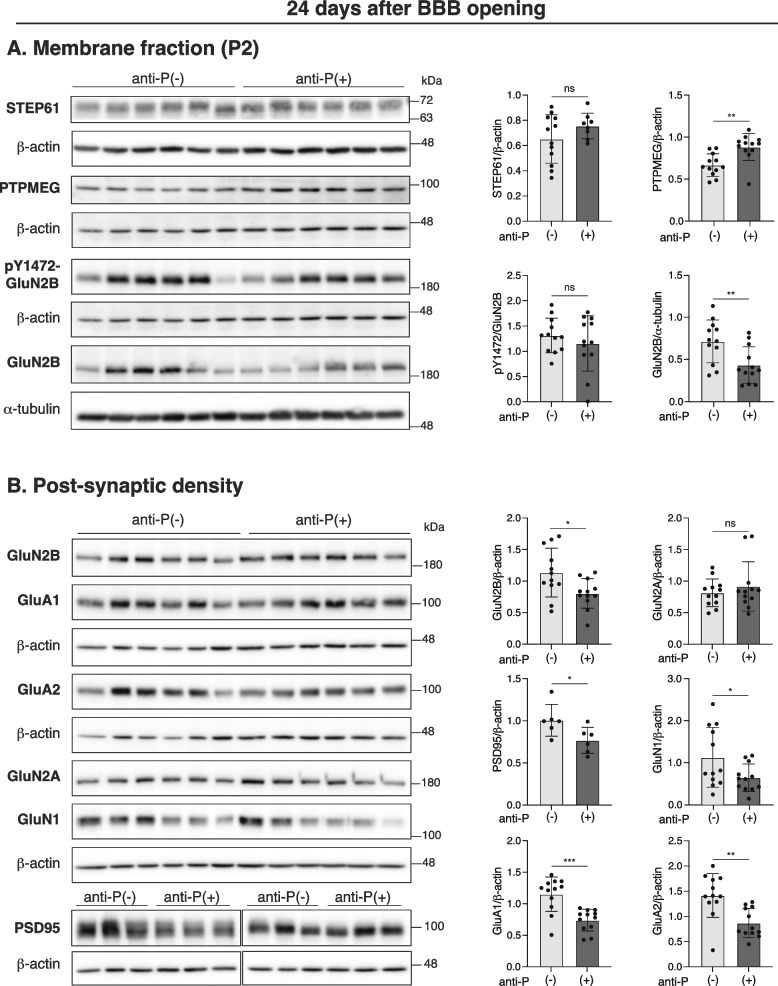
Anti-P effects of synaptosomal membrane and post-synaptic-density (PSD) proteins after 24 days of BBB-breaching with LPS. Immunoblot analysis of the indicated proteins and the corresponding quantification graphs normalized against β-actin. **A** Total synaptosomal membrane fractions (P2) show increased levels of PTPMEG without changes in phosphorylated GluN2B Tyr1472 (pY1472) in anti-P(+) compared with anti-P(-) mice (*n* = 12 per group; mean ± SD; **P < 0.01, unpaired t-test). STEP61 is similar among the two groups. **B** Post-synaptic density (PSD) fractions. NMDAR (GluN2B, GluN2A and GluN1) and AMPAR (GluA1 and GluA2) subunits (*n* = 12 per group; mean ± SD; *P < 0.05, **P < 0.01, ***P < 0.001, unpaired t-test). PSD-95 also decreased in PSD from anti-P(+) mice (*n* = 6 per group; mean ± SD; *P < 0.05, unpaired t-test). All proteins were normalized to their corresponding beta-actin bands. pY1472-GluN2B was additionally normalized against GluN2B total (previously normalized for actin)

Analysis of the same synaptic proteins 24 days after LPS treatment showed that PTPMEG levels remained elevated in anti-P(+) mice (Fig. 4A). Notably, his sustained increase in PTPMEG now coincided not only with reduced NMDAR levels, as previously observed in NSPA-KO mice (Espinoza et al. 2020), but also with decreased AMPAR subunit levels (Fig. 4B). At this later time point, pTyr1472-GluN2B levels were similar to those in anti-P(-) control mice (Fig. 4A). In contrast to the previous 10-day time point, PSD-95 levels in the PSD were also reduced in anti-P(+) mice (Fig. 4B). These findings indicate that the long-term effects of anti-P include progressive alterations in the composition of excitatory synapses in the hippocampus, detectable up to four weeks after a transient BBB breach and likely contributing to spatial memory impairment.

### Long-term effects of anti-P antibodies compromise microglia and neuronal structure in the hippocampus

**Fig. 5:**
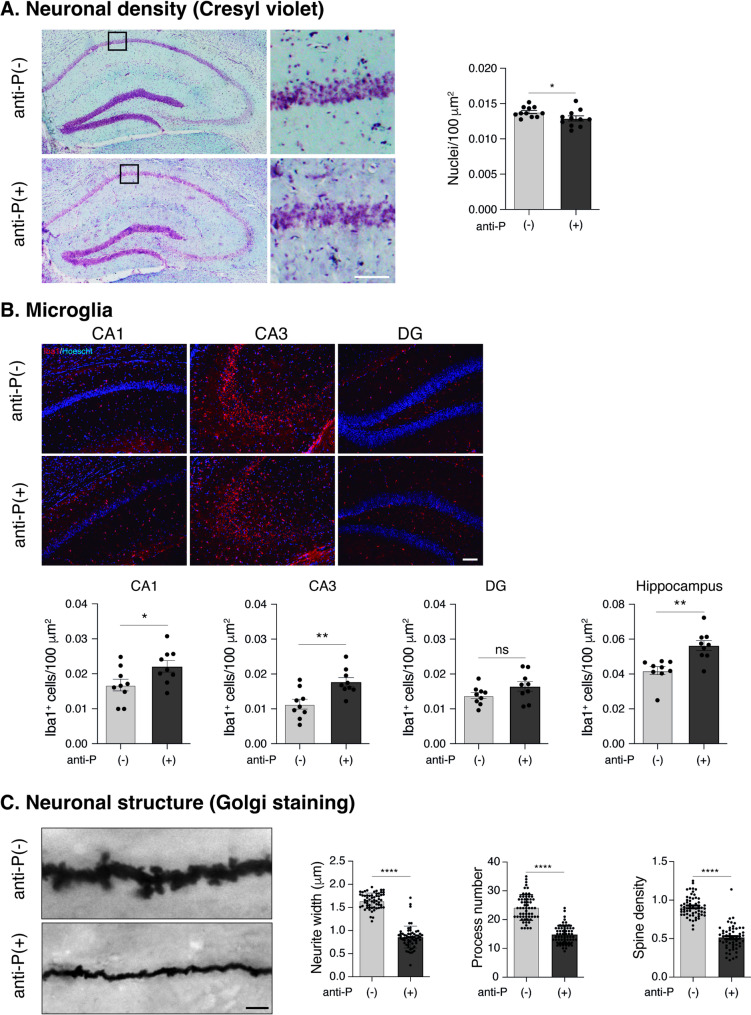
Anti-P long-term effects include minor neuronal loss, increased microglia and decreased dendritic spines. Representative images of hippocampal sections (A. and B.) and Golgi-stained dendrites (**C**) from the brains of anti-P(+) and anti-P(-) mice after 24 days of BBB-breaching with LPS. Graphs show the corresponding quantifications. **A** Cresyl violet staining section showing ~ 7% decreased neuronal nuclei number (*n* = 3 per group; Mean ± SD; *P < 0.05, unpaired t-test). **B** Iba1 staining in hippocampal CA1, CA3 and dentate gyrus (DG) show increased microglia population (*n* = 3 per group; Mean ± SD; *P < 0.05, **P < 0.01, unpaired t-test). C Maximal projections of neurite stereoscopic images reveal decreased neurite width, process number and spine densities (*n* = 3 per group; Mean ± SD; ****P < 0.0001, unpaired t-test)

**Fig. 6:**
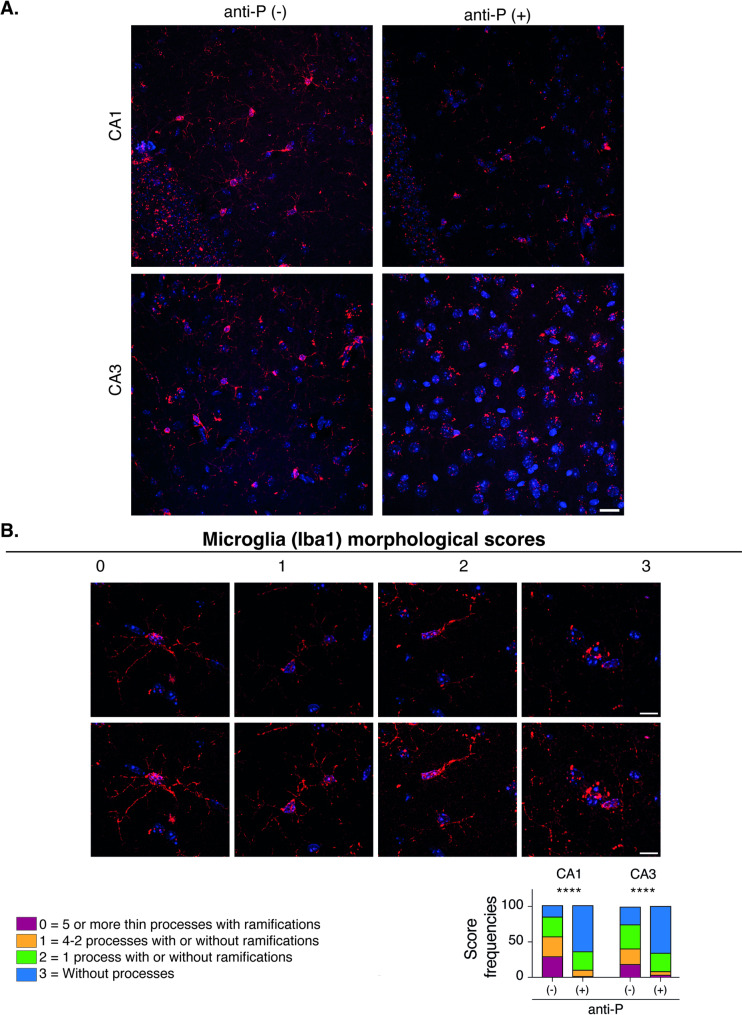
Anti-P long-term effects reduces the morphological ramifications of microglial cells. **A** Images of microglia Iba1(+) cells in maximal projections of confocal images taken in CA1 and CA3 hippocampal regions; **B** Microglial cells representative morphologies after deconvolution and maximal projection showing the scores used for their classification (Scale bar = 20 μm). The contingency graph depicting score frequencies shows a distribution change toward a morphology with less ramifications in the anti-P(+) mice (****p = 0,0001, Chi-square; *n* = 3 mice in each group; Nº of analyzed cells: Anti-P(-), CA1 = 105 cells; CA3 = 154 cells; Anti-P(+) CA1 = 163 cells; CA3 = 128 cells)

Histochemistry analysis of the hippocampus 24 days after LPS treatment revealed ~ 7% neuronal loss in anti-P(+) mice, as shown by Cresyl Violet staining (Fig. 5A). Immunofluorescence using the Iba1 marker in CA1, CA3 and dentate gyrus (DG) regions, showed an increased number of microglial cells in anti-P(+) mice (Fig. 5B). Golgi-staining, used to assess neuronal morphology (Nestor et al. 2018; Carroll et al. 2024), revealed reduced neurite width, fewer neuronal processes, and decreased synaptic spine density in the hippocampus of anti-P(+) mice (Fig. 5C). Since microglial cells typically transition from a highly ramified to a more ameboid morphology reflecting functional activation (Schafer et al. 2024; Paolicelli et al. 2011), we analyzed whether anti-P affected microglial morphology. Based on described protocols (Nestor et al. 2018), we defined simplified scores to assess the number and complexity of extended processes (neurites). Confocal images revealed reduced ramification of microglial cells in anti-P(+) mice compared with anti-P(-) controls (Fig. 6).

Together, these results indicate that the long-term effects of anti-P include a modest but significant loss of hippocampal neurons, alterations in dendritic architecture with synaptic spine loss, and microglia response characterized by increased cell number and morphological transformation.

## Discussion

This study provides new insights into the pathogenic mechanisms by which anti-P antibodies may disrupt synaptic transmission and contribute to cognitive dysfunction in NPSLE patients. Experiments in primary hippocampal neurons demonstrate that anti-P reduce the surface expression and recycling of excitatory glutamatergic receptors at dendritic spines, key components of synaptic plasticity. These acute effects may underlie the previously reported inhibition of LTP in hippocampal slices and early memory impairment following anti-P exposure (Segovia-Miranda et al. 2015). In parallel, analysis of long-term effects using a P0-immunized mouse model revealed sustained alterations in synaptic components and neuronal structure, in association with spatial memory impairment. The accompanying microglial changes suggest a potential deleterious contribution of microglial activity in the observed neuronal alterations. Together, these findings highlight promising directions for further mechanistic studies and therapeutic exploration, particularly focusing on the roles of PTPMEG and microglia.

Our previous studies on the acute effects of anti-P antibodies showed activation of NMDAR and AMPAR, along with impaired induction of hippocampal LTP at the CA3–CA1 synapses (Segovia-Miranda et al. 2015). Notably, activation of these receptors has been shown to trigger their own endocytosis (Groc and Choquet 2020; Choquet and Opazo 2022), whereas LTP involves an increased plasma membrane insertion of both AMPARs (Groc and Choquet 2020) and GluN2A-containing NMDARs (Yong et al. 2021), likely reflecting their increased recycling at the synapse. Based on this, we investigated whether anti-P affect the cell surface pool of NMDARs and AMPARs. We used hippocampal neurons cultured for 21–24 days in the presence of glial cells, a condition that ensures mature dendritic spine development and promotes NMDAR subunit composition characteristic of the adult hippocampus, where GluN2A expression predominates (Bustos et al. 2014; Al-Hallaq et al. 2007; Hansen et al. 2021). Our results show that anti-P reduce the cell surface levels of GluN2A and GluA1 subunits, while total receptor levels remain unchanged. Combined with the previously described anti-P-induced glutamatergic activation (Segovia-Miranda et al. 2015), these results suggest that anti-P may promote the endocytic removal of these receptors from the plasma membrane. However, both NMDAR and AMPAR undergo continuous cycles of endocytosis and recycling leading to variable and regulated stays at the synapse (Groc and Choquet 2020). The stability and plasticity of their synaptic pools depend on a dynamic equilibrium between lateral diffusion at the cell surface, internalization, and trafficking along endocytic pathways, which can lead to either recycling or degradation (Groc and Choquet 2020; Storey et al. 2025; Yang et al. 2022. To specifically assess receptor recycling at the synaptic compartment, we performed FRAP experiments using SEP-GluN2A and SEP-GluN2B constructs. Synaptic FRAP experiments have previously described a biphasic FRAP pattern (Tatavarty et al. 2013), with a fast component within the first 5 min, likely reflecting lateral diffusion and recycling events within the synapse, and a slower component that reaches a plateau between 20–50 min, probably due to lateral diffusion and recycling from extrasynaptic regions. To minimize the contribution of extrasynaptic diffusion and recycling, we bleached large dendritic segments (50–100 µm) and focused the analysis on individual dendritic spines within the initial 5 min window. Under these conditions, we found that anti-P markedly reduces the fast component of FRAP at dendritic spines, suggesting impaired recycling from intraspinal endocytic compartments. In contrast, the slower FRAP recovery component showed no significant difference between anti-P-treated and control cells. Given that NMDAR trafficking regulates AMPAR trafficking and LTP, that its inhibition disrupts synaptic plasticity associated with memory (Yang et al. 2022), and that a cell surface pool of GluA1 is critically required for LTP induction (Granger et al. 2013), we can reasonably conclude that a reduced cell surface expression of GluN2A and GluA1, as well as the inhibition of GluN2A/2B recycling in dendritic spines, contribute to the previously reported LTP inhibition by anti-P (Segovia-Miranda et al. 2015). These acute effects of anti-P may also underlie the memory impairment observed within 2–6 days after a LPS-induced BBB disruption, which enables circulating anti-P to reach the hippocampus (Segovia-Miranda et al. 2015).

However, cognitive dysfunction in SLE patients can persist even after systemic remission (Kello et al. 2019). It is therefore important to investigate the consequences of prolonged anti-P exposure beyond the transient BBB permeability induced by LPS (Chang et al. 2015; Laflamme et al. 2001). Early studies showed that anti-P generated by immunization with ribosomal proteins are indistinguishable from those found in SLE patients in terms of fine specificity for 11-mer synthetic P peptides (Elkon et al. 1988). To study long-term effects, we immunized mice with recombinant P0 ribosomal protein, previously shown to elicit anti-P (Ben-Ami Shor et al. 2014). Serum analysis by ELISA and immunoblot assays using competition with 11-mer synthetic P peptide and a truncated version of P0 lacking the P epitope, confirmed that P0-immunized C57BL/6 mice predominantly produced anti-P. To compare the effects of actively generated antibodies with those observed in our earlier passive transfer model (Bravo-Zehnder et al. 2015), we applied the same spatial memory flexibility test.

Remarkably, we observed similar memory impairments at both 10 and 24 days after LPS-induced BBB breaching. Since BBB integrity has been reported to recover within ~ 48 h post-LPS (Chang et al. 2015; Laflamme et al. 2001), and circulating antibodies are no longer detectable in the hippocampus after two weeks (Chang et al. 2015), these results suggest that anti-P induce persistent neuronal dysfunction beyond their immediate presence into the brain. This experimental model enabled us to examine hippocampal alterations guided by previously reported synaptic changes in the NSPA-KO mice (Segovia-Miranda et al. 2015; Espinoza et al. 2020), and histological and neuronal structural abnormalities described in the DNRab-expressing mice model (Nestor et al. 2018; Carroll et al. 2024).

We previously showed that NSPA, the neuronal surface protein cross-reacting with anti-P, functions as an E3-ubiquitin ligase that regulates synaptic activity and NMDAR distribution, involving the tyrosine phosphatase PTPMEG as one of its substrates (Segovia-Miranda et al. 2015; Espinoza et al. 2020). Phosphorylation critically controls the cell surface stability and PSD localization of NMDAR and AMPAR, with tyrosine phosphatases such as PTPMEG and STEP61 acting as negative regulators (Espinoza et al. 2020; Won and Roche 2021; Kohda et al. 2013a). NSPA-KO mice exhibit memory impairment associated with decreased levels of GluN2A and GluN2B in PSD fractions, reduced phosphorylation of GluN2B at Tyr1472, and increased PTPMEG levels in synaptosomal membrane fractions (Espinoza et al. 2020). In contrast, AMPAR and PSD-95 levels in PSD fractions, as well as STEP61 levels in membrane fractions, remain unchanged in NSPA-KO compared with WT controls (Espinoza et al. 2020). GluN2B-Tyr1472 conforms an endocytic signal that is blocked by phosphorylation, while its dephosphorylation promotes internalization and reduces NMDAR retention at the PSD (Snyder et al. 2005). Based on these observations, we examined these parameters at 10 and 24 days after LPS treatment and found that anti-P elicit effects beyond the alterations seen in the NSPA-KO mice.

Anti-P(+) mice exhibited consistently elevated levels of membrane-associated PTPMEG in the hippocampus at both 10 and 24 days post-BBB-breaching, while other Changes varied over time. At 10 days post-LPS i.p. injection, anti-P exposure led to reduced phosphorylation of GluN2B at Tyr1472, consistent with increased PTPMEG levels. However, at this time point, we did not observe significant changes in NMDAR or AMPAR levels in the PSD fraction. Interestingly, PSD-95 levels were increased, suggesting a possible compensatory mechanism aimed at stabilizing and retaining glutamatergic receptors at synapses, given the known scaffolding role of PSD-95 in organizing receptors and other components at excitatory synapses (Won et al. 2017; Zhu et al. 2016). Despite this apparent compensation, anti-P(+) mice still showed impaired performance in the memory flexibility test. The reduced phosphorylation of GluN2B-Tyr1472 may have compromised NMDAR trafficking, which is critical for synaptic plasticity (Yang et al. 2022). On the other hand, further alterations associated with memory impairment emerged after 24 days of BBB breaching. Although PTPMEG levels remained elevated in membrane fractions, GluN2B-Tyr1472 phosphorylation had returned to control levels. Notably, however, the PSD now showed a marked reduction in multiple components, encompassing NMDAR subunits (GluN1 and GluN2B), AMPAR subunits (GluA1 and GluA2), and PDS-95. These widespread losses strongly suggest a breakdown of excitatory synaptic integrity, which likely contributes to the sustained spatial memory impairment observed at this stage.

The increased levels of PTPMEG observed in anti-P(+) mice mimic the phenotype of NSPA-KO mice (Espinoza et al. 2020), suggesting that anti-P may inhibit NSPA’s role in regulating this tyrosine phosphatase, which is involved in stabilizing NMDAR at the synapse (Espinoza et al. 2020). Tyrosine phosphorylation critically regulates the trafficking and retention of NMDARs and AMPARs at the PSD, playing a central role in synaptic plasticity (Won and Roche 2021; Groc and Choquet 2020; Prybylowski et al. 2005; Xu et al. 2015; Trepanier et al. 2012). While the tyrosine phosphatase STEP61 has been primarily implicated in this process (Won and Roche 2021), synaptic membrane fractions from NSPA-KO (Espinoza et al. 2020) and anti-P(+) mice showed unaltered STEP61 levels. Although less studied, PTPMEG likely contributes to this pathway by dephosphorylating GluN2B-MNDAR and GluA2-AMPAR subunits (Espinoza et al. 2020; Kohda et al. 2013a; Hironaka et al. 2000). Dephosphorylation of GluN2B-Tyr1472 promotes NMDAR endocytosis and reduces their retention at the PSD (Prybylowski et al. 2005; Xu et al. 2006), a process previously associated to the elevated PTPMEG levels and memory impairment in NSPA-KO mice (Espinoza et al. 2020). Similarly, GluA2-Y876 dephosphorylation promotes GluA2 AMPAR endocytosis associated with long-term-depression in cerebellum and hippocampus (Kohda et al. 2013a, 2013b; Ahmadian et al. 2004; Moult et al. 2006; Scholz et al. 2010).

PTPMEG is highly expressed in the brain (Szczaluba et al. 2018; Gu and Majerus 1996) and several studies suggest it plays essential roles in neuronal function (Barake et al. 2022; Szczaluba et al. 2018; Williamson et al. 2015; Kohda et al. 2013a; Kina et al. 2007). For instance, neurodevelopmental disorders in the Rett syndrome are mostly caused by mutations in MECP2, a transcription factor that regulates PTPMEG expression (Williamson et al. 2015). An atypical Rett case involved a deletion of the PTPMEG gene itself (Williamson et al. 2015), and MECP2-KO mice exhibit impaired hippocampal synaptic plasticity and memory (Asaka et al. 2006; Moretti et al. 2006). PTPMEG is targeted to the plasma membrane and cytoskeleton via its FERM domain and distributes broadly in neurons, including dendritic spines in hippocampal neurons (Szczaluba et al. 2018). A point mutation within the FERM domain, reported in a patient with autistic features and hypotonia, prevents its location to dendritic spines (Szczaluba et al. 2018). Other studies have shown that cerebellar motor learning and long-term-depression (LTD) depend on PTPMEG-mediated Tyr dephosphorylation of AMPAR subunits (Kohda et al. 2013a; Kina et al. 2007). Here, we found elevated levels of PTPMEG in synaptosomal membranes even before the reduction of PSD components involved in synaptic plasticity. This finding, together with the phenotype of NSPA-KO mice (Espinoza et al. 2020), point to PTPMEG as a likely driver of the synaptic alterations associated with memory impairment promoted by anti-P. Interestingly, alendronate, a drug currently used to treat osteoporosis (Al Lawati et al. 2025), is a potent inhibitor of PTPMEG (Opas et al. 1997) and has been shown to access the brain via systemic (Cibickova et al. 2007) or nasal administration (Zameer et al. 2021). As such, PTPMEG represents a potential therapeutic target, and it will be important to investigate whether alendronate can counteract the effects of anti-P.

PSD-95, another synaptic component affected by anti-P, is a scaffold protein essential for synaptic plasticity (Zhu et al. 2016). It organizes a complex network that stabilizes and regulates synaptic function through interactions with synaptic proteins, the cytoskeleton, and ion channels (Zhu et al. 2016; Coley and Gao 2018; Chen et al. 2015; Matt et al. 2018). PSD-95 directly binds to NMDA-GluN2 subunits as well as to TARP auxiliary subunits of AMPARs, promoting the clustering and stabilization of these receptors at the PSD (Daly et al. 2025; Shen et al. 2023; Opazo et al. 2012). The integrity of the PSD structure is highly dependent on the regulated concentration of PSD-95 (Shen et al. 2023), which itself is sensitive to changes in other synaptic components, such as NMDARs (Zhu et al. 2016; Compans et al. 2021). Neuropathogenic conditions derived from alterations in synapse morphology and function frequently involve dysfunctions or aberrant up- or down-expression of PSD-95 (Won et al. 2017; Bustos et al. 2014; Zhu et al. 2016; Levy et al. 2022). In our study, the reduced levels of NMDARs and AMPARs at the PSD of anti-P(+) mice predict a decreased synaptic strength that could reduce PSD-95 at PSD and may initiate a negative regulatory loop (Compans et al. 2021), wherein insufficient PSD-95 fails to retain receptors and other essential synaptic components at the PSD (Shen et al. 2023).

Finally, additional interesting changes observed in the hippocampus of P0 immunized anti-P(+) mice 24 days post BBB breaching include ~ 7% neuronal loss, reduced dendritic width, decreased spine density in the remaining neurons, as well as increased density of Iba1(+) microglia displaying a less ramified phenotype. These changes may closely resemble those described in the mouse model of DNRab-mediated memory impairment (Nestor et al. 2018), in which DNRabs cause ~ 30% of neuronal loss in the hippocampus, and the surviving neurons initiate a neuroinflammation process that persists for a long time after DNRabs are no longer detectable in the brain (Nestor et al. 2018). The pathogenic mechanism of DNRabs involves synaptic pruning mediated by microglia reactive to high mobility group box protein 1 (HMGB1), a transcription factor released from stressed neurons, which not only stimulates the microglia to secrete C1q, but also interacts with both C1q and NMDAR, thus tagging synapses with C1q for their selective engulfment by the microglia (Nestor et al. 2018). The intertwined action of HMGB1 and C1q engages additional molecular players in a maladaptive feedforward loop of neuroinflammation, sustaining cognitive dysfunction (Nestor et al. 2018; Carroll et al. 2024). Notably, the BBB-permeable angiotensin converting enzyme (ACE) inhibitor, captopril, or the angiotensin-receptor blocker (ARB), telmisartan, have shown efficacy in counteracting this microglia-mediated synaptic pruning, reversing the DNRab-induced memory impairment (Nestor et al. 2018; Carroll et al. 2024). Whether similar mechanisms underlie the anti-P-induced reduction of dendritic spine density remains an important question for future investigations. Next experiments using the P0(+) mice model should assess HMGB1 and C1q colocalization with synaptic markers, as well as the counteracting effects of ACE or ARB inhibitors, as described in the DNRab mice model (Nestor et al. 2018; Carroll et al. 2024). Such studies could help determine whether microglia contribute to anti-P pathogenicity and may broaden the therapeutic relevance of these strategies to include anti-P(+) patients, in addition to those harboring DNRabs.

## Conclusions

This study advances our understanding of the pathogenicity of anti-ribosomal P antibodies (anti-P) associated with cognitive dysfunction in NPSLE. We demonstrate that these antibodies exert acute effects on hippocampal neurons in culture, reducing the trafficking of NMDAR at dendritic spines and the surface levels of both NMDAR and AMPAR, which may account for the previously reported inhibition of LTP at CA3–CA1 synapsis (Segovia-Miranda et al. 2015) and short-term memory impairment in passive transfer experiments in mice (Bravo-Zehnder et al. 2015). In addition, in a mouse model immunized to produce anti-P antibodies, we show that exposure to conditions known to induce a transient opening of the BBB leads to long-lasting spatial memory impairment, associated with alterations in synaptic structure and changes in microglial cell number and morphology. The loss of NMDAR, AMPAR, and PSD-95 at the postsynaptic density (PSD), together with reduced dendritic spine density, very likely underlie the memory impairment. The PSD protein level changes are preceded by increased PTPMEG levels in synaptic membranes, resembling the phenotype of NSPA-deficient mice (Espinoza et al. 2020) and therefore suggesting that anti-P may inhibit the function of its neuronal cell surface cross-reactive protein (Matus et al. 2007). The roles of PTPMEG in the loss of PSD components and of reactive microglia in dendritic spine elimination, potentially through aberrant synaptic pruning, remain important mechanistic questions with therapeutic implications for NPSLE patients harboring anti-P antibodies.

## Supplementary Information

Supplementary Material 1

Supplementary Material 2

Supplementary Material 3

Supplementary Material 4

Supplementary Material 5

## Data Availability

No datasets were generated or analysed during the current study.

## Abbreviations

ACE: Angiotensin-converting enzyme
AMPAR: α-Amino-3-hydroxy-5-methyl-4-isoxazolepropionic acid receptor
BBB: Blood–brain barrier
CA1: Cornu Ammonis
CD: Cognitive Dysfunction
DG: Dentate gyrus
DNRabs: Anti-double-stranded DNA cross-reactive with NMDAR antibodies
LTP: Long-term potentiation
LPS: Lipopolysaccharide
NMDAR: N-methyl- D -aspartate receptor
NPSLE: Neuropsychiatric Systemic Lupus Erythematosus
NSPA: Neuronal surface P antigen
PSD: Postsynaptic density
PTPMEG: Megakaryocyte protein tyrosine phosphatase
Anti-P: Anti-ribosomal P protein- and NSPA-P epitope antibodies
STEP: Striatal-enriched protein tyrosine phosphatase
SLE: Systemic lupus erythematosus

## References

[CR1] Ahmadian G, Ju W, Liu L, Wyszynski M, Lee SH, Dunah AW, et al. Tyrosine phosphorylation of GluR2 is required for insulin-stimulated AMPA receptor endocytosis and LTD. EMBO J. 2004;23(5):1040–50. 14976558 10.1038/sj.emboj.7600126 PMC380981

[CR2] Al Lawati H, Al Busaidi S, Al Rawahi T, Al Lawati A, Kifah A, Das S. Alendronate for effective treatment of male osteoporosis: an insight. Curr Pharm des. 2025;31(1):26–36. 39238374 10.2174/0113816128310838240820065324

[CR3] Al-Hallaq RA, Conrads TP, Veenstra TD, Wenthold RJ. NMDA di-heteromeric receptor populations and associated proteins in rat hippocampus. J Neurosci. 2007;27(31):8334–43. 17670980 10.1523/JNEUROSCI.2155-07.2007 PMC2263005

[CR4] Asaka Y, Jugloff DG, Zhang L, Eubanks JH, Fitzsimonds RM. Hippocampal synaptic plasticity is impaired in the *Mecp2*-null mouse model of Rett syndrome. Neurobiol Dis. 2006;21(1):217–27. 16087343 10.1016/j.nbd.2005.07.005

[CR5] Barake F, Bravo-Zehnder M, Gonzalez A. Progress in the mechanism of neuronal surface P antigen modulating hippocampal function and implications for autoimmune brain disease. Curr Opin Neurol. 2022;35(3):436–42. 35674087 10.1097/WCO.0000000000001054

[CR6] Beattie EC, Carroll RC, Yu X, Morishita W, Yasuda H, von Zastrow M, et al. Regulation of AMPA receptor endocytosis by a signaling mechanism shared with LTD. Nat Neurosci. 2000;3(12):1291–300. 11100150 10.1038/81823

[CR7] Ben-Ami Shor D, Blank M, Reuter S, Matthias T, Beiglass I, Volkov A, et al. Anti-ribosomal-P antibodies accelerate lupus glomerulonephritis and induce lupus nephritis in naive mice. J Autoimmun. 2014. 10.1016/j.jaut.2014.02.013. 24662148 10.1016/j.jaut.2014.02.013

[CR8] Bertsias GK, Boumpas DT. Pathogenesis, diagnosis and management of neuropsychiatric SLE manifestations. Nat Rev Rheumatol. 2010;6(6):358–67. 20458332 10.1038/nrrheum.2010.62

[CR9] Bonfa E, Elkon KB. Clinical and serologic associations of the antiribosomal P protein antibody. Arthritis Rheum. 1986;29(8):981–5. 3527180 10.1002/art.1780290806

[CR10] Bonfa E, Golombek SJ, Kaufman LD, Skelly S, Weissbach H, Brot N, et al. Association between lupus psychosis and anti-ribosomal P protein antibodies. N Engl J Med. 1987;317(5):265–71. 3496538 10.1056/NEJM198707303170503

[CR11] Bravo-Zehnder M, Orio P, Norambuena A, Wallner M, Meera P, Toro L, et al. Apical sorting of a voltage- and Ca2+-activated K+ channel alpha -subunit in Madin-Darby canine kidney cells is independent of N-glycosylation. Proc Natl Acad Sci U S A. 2000;97(24):13114–9. 11069304 10.1073/pnas.240455697 PMC27187

[CR12] Bravo-Zehnder M, Toledo EM, Segovia-Miranda F, Serrano FG, Benito MJ, Metz C, et al. Anti-ribosomal p protein autoantibodies from patients with neuropsychiatric lupus impair memory in mice. Arthritis Rheumatol. 2015;67(1):204–14. 25302407 10.1002/art.38900

[CR13] Bustos FJ, Varela-Nallar L, Campos M, Henriquez B, Phillips M, Opazo C, et al. PSD95 suppresses dendritic arbor development in mature hippocampal neurons by occluding the clustering of NR2B-NMDA receptors. PLoS ONE. 2014;9(4):e94037. 24705401 10.1371/journal.pone.0094037 PMC3976375

[CR14] Cancino J, Torrealba C, Soza A, Yuseff MI, Gravotta D, Henklein P, et al. Antibody to AP1B adaptor blocks biosynthetic and recycling routes of basolateral proteins at recycling endosomes. Mol Biol Cell. 2007;18(12):4872–84. 17881725 10.1091/mbc.E07-06-0563 PMC2096610

[CR15] Carroll KR, Mizrachi M, Simmons S, Toz B, Kowal C, Wingard J, et al. Lupus autoantibodies initiate neuroinflammation sustained by continuous HMGB1:RAGE signaling and reversed by increased LAIR-1 expression. Nat Immunol. 2024;25(4):671–81. 38448779 10.1038/s41590-024-01772-6 PMC11141703

[CR16] Chang EH, Volpe BT, Mackay M, Aranow C, Watson P, Kowal C, et al. Selective impairment of spatial cognition caused by autoantibodies to the N-methyl-D-aspartate receptor. EBioMedicine. 2015;2(7):755–64. 26286205 10.1016/j.ebiom.2015.05.027 PMC4534689

[CR17] Chen G, Chen KS, Knox J, Inglis J, Bernard A, Martin SJ, et al. A learning deficit related to age and beta-amyloid plaques in a mouse model of Alzheimer’s disease. Nature. 2000;408(6815):975–9. 11140684 10.1038/35050103

[CR18] Chen X, Levy JM, Hou A, Winters C, Azzam R, Sousa AA, et al. PSD-95 family MAGUKs are essential for anchoring AMPA and NMDA receptor complexes at the postsynaptic density. Proc Natl Acad Sci U S A. 2015;112(50):E6983–92. 26604311 10.1073/pnas.1517045112 PMC4687590

[CR19] Choquet D, Opazo P. The role of AMPAR lateral diffusion in memory. Semin Cell Dev Biol. 2022;125:76–83. 35123863 10.1016/j.semcdb.2022.01.009

[CR20] Cibickova L, Palicka V, Cibicek N, Cermakova E, Micuda S, Bartosova L, et al. Differential effects of statins and alendronate on cholinesterases in serum and brain of rats. Physiol Res. 2007;56(6):765–70. 17087598 10.33549/physiolres.931121

[CR21] Citri A, Malenka RC. Synaptic plasticity: multiple forms, functions, and mechanisms. Neuropsychopharmacology. 2008;33(1):18–41. 17728696 10.1038/sj.npp.1301559

[CR22] Coley AA, Gao WJ. PSD95: a synaptic protein implicated in schizophrenia or autism? Prog Neuropsychopharmacol Biol Psychiatry. 2018;82:187–94. 29169997 10.1016/j.pnpbp.2017.11.016 PMC5801047

[CR23] Compans B, Camus C, Kallergi E, Sposini S, Martineau M, Butler C, et al. NMDAR-dependent long-term depression is associated with increased short term plasticity through autophagy mediated loss of PSD-95. Nat Commun. 2021;12(1):2849. 33990590 10.1038/s41467-021-23133-9 PMC8121912

[CR24] Daly S, Bulovaite E, Handa A, Morris K, Muresan L, Adams C, et al. 3D super-resolution imaging of PSD95 reveals an abundance of diffuse protein supercomplexes in the mouse brain. ACS Chem Neurosci. 2025;16(1):40–51. 39702971 10.1021/acschemneuro.4c00684 PMC11697326

[CR25] Dupuis JP, Nicole O, Groc L. *NMDA* receptor functions in health and disease: old actor, new dimensions. Neuron. 2023;111(15):2312–28. 37236178 10.1016/j.neuron.2023.05.002

[CR26] Elkon K, Bonfa E, Llovet R, Danho W, Weissbach H, Brot N. Properties of the ribosomal P2 protein autoantigen are similar to those of foreign protein antigens. Proc Natl Acad Sci U S A. 1988;85(14):5186–9. 2455896 10.1073/pnas.85.14.5186 PMC281713

[CR27] Espinoza S, Arredondo SB, Barake F, Carvajal F, Guerrero FG, Segovia-Miranda F, et al. Neuronal surface P antigen (NSPA) modulates postsynaptic NMDAR stability through ubiquitination of tyrosine phosphatase PTPMEG. BMC Biol. 2020;18(1):164. 33158444 10.1186/s12915-020-00877-2 PMC7648380

[CR28] Gaburo N Jr, de Carvalho JF, Timo-Iaria CIM, Bueno C, Reichlin M, Viana VS, et al. Electrophysiological dysfunction induced by anti-ribosomal P protein antibodies injection into the lateral ventricle of the rat brain. Lupus. 2017;26(5):463–9. 28394228 10.1177/0961203316666185

[CR29] Gonzalez A, Massardo L. Antibodies and the brain: antiribosomal P protein antibody and the clinical effects in patients with systemic lupus erythematosus. Curr Opin Neurol. 2018;31(3):300–5. 29461425 10.1097/WCO.0000000000000549

[CR30] Govoni M, Hanly JG. The management of neuropsychiatric lupus in the 21st century: still so many unmet needs? Rheumatology (Oxford). 2020;59(Suppl5):v52–62. 33280014 10.1093/rheumatology/keaa404 PMC7719041

[CR31] Granger AJ, Shi Y, Lu W, Cerpas M, Nicoll RA. LTP requires a reserve pool of glutamate receptors independent of subunit type. Nature. 2013;493(7433):495–500. 23235828 10.1038/nature11775 PMC3998843

[CR32] Groc L, Choquet D. Linking glutamate receptor movements and synapse function. Science. 2020. 10.1126/science.aay4631. 32527803 10.1126/science.aay4631

[CR33] Gu M, Majerus PW. The properties of the protein tyrosine phosphatase PTPMEG. J Biol Chem. 1996;271(44):27751–9. 8910369 10.1074/jbc.271.44.27751

[CR34] Hansen KB, Wollmuth LP, Bowie D, Furukawa H, Menniti FS, Sobolevsky AI, et al. Structure, function, and pharmacology of glutamate receptor ion channels. Pharmacol Rev. 2021;73(4):298–487. 34753794 10.1124/pharmrev.120.000131 PMC8626789

[CR35] Hironaka K, Umemori H, Tezuka T, Mishina M, Yamamoto T. The protein-tyrosine phosphatase PTPMEG interacts with glutamate receptor delta 2 and epsilon subunits. J Biol Chem. 2000;275(21):16167–73. 10748123 10.1074/jbc.M909302199

[CR36] Huerta PT, Kowal C, DeGiorgio LA, Volpe BT, Diamond B. Immunity and behavior: antibodies alter emotion. Proc Natl Acad Sci U S A. 2006;103(3):678–83. 16407105 10.1073/pnas.0510055103 PMC1334673

[CR37] Kaech S, Banker G. Culturing hippocampal neurons. Nat Protoc. 2006;1(5):2406–15. 17406484 10.1038/nprot.2006.356

[CR38] Katzav A, Solodeev I, Brodsky O, Chapman J, Pick CG, Blank M, et al. Induction of autoimmune depression in mice by anti-ribosomal P antibodies via the limbic system. Arthritis Rheum. 2007;56(3):938–48. 17328071 10.1002/art.22419

[CR39] Katzav A, Ben-Ziv T, Chapman J, Blank M, Reichlin M, Shoenfeld Y. Anti-P ribosomal antibodies induce defect in smell capability in a model of CNS -SLE (depression). J Autoimmun. 2008;31(4):393–8. 18947972 10.1016/j.jaut.2008.09.002

[CR40] Kello N, Anderson E, Diamond B. Cognitive Dysfunction in SLE: a case for initiating trials. Arthritis Rheumatol. 2019. 10.1002/art.40933 PMC6716992 31102496

[CR41] Kina S, Tezuka T, Kusakawa S, Kishimoto Y, Kakizawa S, Hashimoto K, et al. Involvement of protein-tyrosine phosphatase PTPMEG in motor learning and cerebellar long-term depression. Eur J Neurosci. 2007;26(8):2269–78. 17953619 10.1111/j.1460-9568.2007.05829.x

[CR42] Kohda K, Kakegawa W, Matsuda S, Yamamoto T, Hirano H, Yuzaki M. The delta2 glutamate receptor gates long-term depression by coordinating interactions between two AMPA receptor phosphorylation sites. Proc Natl Acad Sci U S A. 2013a;110(10):E948–57. 23431139 10.1073/pnas.1218380110 PMC3593918

[CR43] Kohda K, Kakegawa W, Yuzaki M. Unlocking the secrets of the delta2 glutamate receptor: a gatekeeper for synaptic plasticity in the cerebellum. Commun Integr Biol. 2013b;6(6):e26466. 24563706 10.4161/cib.26466 PMC3916355

[CR44] Kopec CD, Li B, Wei W, Boehm J, Malinow R. Glutamate receptor exocytosis and spine enlargement during chemically induced long-term potentiation. J Neurosci. 2006;26(7):2000–9. 16481433 10.1523/JNEUROSCI.3918-05.2006 PMC6674938

[CR45] Koren E, Reichlin MW, Koscec M, Fugate RD, Reichlin M. Autoantibodies to the ribosomal P proteins react with a plasma membrane- related target on human cells. J Clin Invest. 1992;89(4):1236–41. 1313450 10.1172/JCI115707 PMC442983

[CR46] Kowal C, DeGiorgio LA, Nakaoka T, Hetherington H, Huerta PT, Diamond B, et al. Cognition and immunity; antibody impairs memory. Immunity. 2004;21(2):179–88. 15308099 10.1016/j.immuni.2004.07.011

[CR47] Kowal C, Degiorgio LA, Lee JY, Edgar MA, Huerta PT, Volpe BT, et al. Human lupus autoantibodies against NMDA receptors mediate cognitive impairment. Proc Natl Acad Sci USA. 2006;103(52):19854–9. 17170137 10.1073/pnas.0608397104 PMC1702320

[CR48] Laflamme N, Soucy G, Rivest S. Circulating cell wall components derived from gram-negative, not gram-positive, bacteria cause a profound induction of the gene-encoding Toll-like receptor 2 in the CNS. J Neurochem. 2001;79(3):648–57. 11701768 10.1046/j.1471-4159.2001.00603.x

[CR49] Levy AM, Gomez-Puertas P, Tumer Z. Neurodevelopmental disorders associated with PSD-95 and its interaction partners. Int J Mol Sci. 2022. 10.3390/ijms23084390. 35457207 10.3390/ijms23084390 PMC9025546

[CR50] Lombroso PJ, Ogren M, Kurup P, Nairn AC. Molecular underpinnings of neurodegenerative disorders: striatal-enriched protein tyrosine phosphatase signaling and synaptic plasticity. F1000Res. 2016;5. 10.12688/f1000research.8571.1 PMC5642311 29098072

[CR51] Mahler M, Kessenbrock K, Raats J, Williams R, Fritzler MJ, Bluthner M. Characterization of the human autoimmune response to the major C-terminal epitope of the ribosomal P proteins. J Mol Med. 2003;81(3):194–204. 12682728 10.1007/s00109-003-0423-1

[CR52] Massardo L, Metz C, Pardo E, Mezzano V, Babul M, Jarpa E, et al. Autoantibodies against galectin-8: their specificity, association with lymphopenia in systemic lupus erythematosus and detection in rheumatoid arthritis and acute inflammation. Lupus. 2009;18(6):539–46. 19395456 10.1177/0961203308099973

[CR53] Massardo L, Bravo-Zehnder M, Calderon J, Flores P, Padilla O, Aguirre JM, et al. Anti-N-methyl-D-aspartate receptor and anti-ribosomal-P autoantibodies contribute to cognitive dysfunction in systemic lupus erythematosus. Lupus. 2015;24(6):558–68. 25318968 10.1177/0961203314555538

[CR54] Matt L, Kim K, Hergarden AC, Patriarchi T, Malik ZA, Park DK, et al. alpha-Actinin Anchors PSD-95 at Postsynaptic Sites. Neuron. 2018;97(5):1094-109 e9. 29429936 10.1016/j.neuron.2018.01.036 PMC5963734

[CR55] Matus S, Burgos PV, Bravo-Zehnder M, Kraft R, Porras OH, Farias P, et al. Antiribosomal-P autoantibodies from psychiatric lupus target a novel neuronal surface protein causing calcium influx and apoptosis. J Exp Med. 2007;204(13):3221–34. 18056288 10.1084/jem.20071285 PMC2150977

[CR56] Megias M, Emri Z, Freund TF, Gulyas AI. Total number and distribution of inhibitory and excitatory synapses on hippocampal CA1 pyramidal cells. Neuroscience. 2001;102(3):527–40. 11226691 10.1016/s0306-4522(00)00496-6

[CR57] Moretti P, Levenson JM, Battaglia F, Atkinson R, Teague R, Antalffy B, et al. Learning and memory and synaptic plasticity are impaired in a mouse model of Rett syndrome. J Neurosci. 2006;26(1):319–27. 16399702 10.1523/JNEUROSCI.2623-05.2006 PMC6674314

[CR58] Moult PR, Gladding CM, Sanderson TM, Fitzjohn SM, Bashir ZI, Molnar E, et al. Tyrosine phosphatases regulate AMPA receptor trafficking during metabotropic glutamate receptor-mediated long-term depression. J Neurosci. 2006;26(9):2544–54. 16510732 10.1523/JNEUROSCI.4322-05.2006 PMC6793648

[CR59] Nestor J, Arinuma Y, Huerta TS, Kowal C, Nasiri E, Kello N, et al. Lupus antibodies induce behavioral changes mediated by microglia and blocked by ACE inhibitors. J Exp Med. 2018;215(10):2554–66. 30185634 10.1084/jem.20180776 PMC6170183

[CR60] Neves G, Cooke SF, Bliss TV. Synaptic plasticity, memory and the hippocampus: a neural network approach to causality. Nat Rev Neurosci. 2008;9(1):65–75. 18094707 10.1038/nrn2303

[CR61] Oakes SG, Martin WJ 2nd, Lisek CA, Powis G. Incomplete hydrolysis of the calcium indicator precursor fura-2 pentaacetoxymethyl ester (fura-2 AM) by cells. Anal Biochem. 1988;169(1):159–66. 3369679 10.1016/0003-2697(88)90267-9

[CR62] Opas EE, Rutledge SJ, Golub E, Stern A, Zimolo Z, Rodan GA, et al. Alendronate inhibition of protein-tyrosine-phosphatase-meg1. Biochem Pharmacol. 1997;54(6):721–7. 9310349 10.1016/s0006-2952(97)00225-6

[CR63] Opazo P, Sainlos M, Choquet D. Regulation of AMPA receptor surface diffusion by PSD-95 slots. Curr Opin Neurobiol. 2012;22(3):453–60. 22051694 10.1016/j.conb.2011.10.010

[CR64] Paolicelli RC, Bolasco G, Pagani F, Maggi L, Scianni M, Panzanelli P, et al. Synaptic pruning by microglia is necessary for normal brain development. Science. 2011;333(6048):1456–8. 21778362 10.1126/science.1202529

[CR65] Petrini EM, Lu J, Cognet L, Lounis B, Ehlers MD, Choquet D. Endocytic trafficking and recycling maintain a pool of mobile surface AMPA receptors required for synaptic potentiation. Neuron. 2009;63(1):92–105. 19607795 10.1016/j.neuron.2009.05.025 PMC2847611

[CR66] Pozzi D, Ban J, Iseppon F, Torre V. An improved method for growing neurons: comparison with standard protocols. J Neurosci Methods. 2017;280:1–10. 28137433 10.1016/j.jneumeth.2017.01.013

[CR67] Prybylowski K, Chang K, Sans N, Kan L, Vicini S, Wenthold RJ. The synaptic localization of NR2B-containing NMDA receptors is controlled by interactions with PDZ proteins and AP-2. Neuron. 2005;47(6):845–57. 16157279 10.1016/j.neuron.2005.08.016 PMC1350965

[CR68] Salazar G, Gonzalez A. Novel mechanism for regulation of epidermal growth factor receptor endocytosis revealed by protein kinase A inhibition. Mol Biol Cell. 2002;13(5):1677–93. 12006662 10.1091/mbc.01-08-0403 PMC111136

[CR69] Santana PA, Alvarez CA, Guzman F, Mercado L. Development of a sandwich ELISA for quantifying hepcidin in rainbow trout. Fish Shellfish Immunol. 2013;35(3):748–55. 23791861 10.1016/j.fsi.2013.06.005

[CR70] Schafer DP, Stevens B, Bennett ML, Bennett FC. Role of Microglia in Central Nervous System Development and Plasticity. Cold Spring Harb Perspect Biol. 2024. 10.1101/cshperspect.a041810 PMC12487714 39349311

[CR71] Scholz R, Berberich S, Rathgeber L, Kolleker A, Kohr G, Kornau HC. AMPA receptor signaling through BRAG2 and Arf6 critical for long-term synaptic depression. Neuron. 2010;66(5):768–80. 20547133 10.1016/j.neuron.2010.05.003

[CR72] Schwartz N, Stock AD, Putterman C. Neuropsychiatric lupus: new mechanistic insights and future treatment directions. Nat Rev Rheumatol. 2019;15(3):137–52. 30659245 10.1038/s41584-018-0156-8 PMC8023338

[CR73] Segovia-Miranda F, Serrano F, Dyrda A, Ampuero E, Retamal C, Bravo-Zehnder M, et al. Pathogenicity of lupus anti-ribosomal p antibodies: role of cross-reacting neuronal surface p antigen in glutamatergic transmission and plasticity in a mouse model. Arthritis Rheumatol. 2015;67(6):1598–610. 25709106 10.1002/art.39081

[CR74] Shahar O, Botvinnik A, Shwartz A, Lerer E, Golding P, Buko A, et al. Effect of chemically synthesized psilocybin and psychedelic mushroom extract on molecular and metabolic profiles in mouse brain. Mol Psychiatry. 2024;29(7):2059–73. 38378926 10.1038/s41380-024-02477-w PMC11408259

[CR75] Shen Z, Sun D, Savastano A, Varga SJ, Cima-Omori MS, Becker S, et al. Multivalent tau/PSD-95 interactions arrest in vitro condensates and clusters mimicking the postsynaptic density. Nat Commun. 2023;14(1):6839. 37891164 10.1038/s41467-023-42295-2 PMC10611757

[CR76] Snyder EM, Nong Y, Almeida CG, Paul S, Moran T, Choi EY, et al. Regulation of NMDA receptor trafficking by amyloid-beta. Nat Neurosci. 2005;8(8):1051–8. 16025111 10.1038/nn1503

[CR77] Spruston N. Pyramidal neurons: dendritic structure and synaptic integration. Nat Rev Neurosci. 2008;9(3):206–21. 18270515 10.1038/nrn2286

[CR78] Storey GP, Riquelme R, Barria A. Activity-dependent internalization of Glun2B-containing NMDARs is required for synaptic incorporation of Glun2A and synaptic plasticity. J Neurosci. 2025. 10.1523/JNEUROSCI.0823-24.2024. 39562042 10.1523/JNEUROSCI.0823-24.2024 PMC11756629

[CR79] Szczaluba K, Chmielewska JJ, Sokolowska O, Rydzanicz M, Szymanska K, Feleszko W, et al. Neurodevelopmental phenotype caused by a de novo PTPN4 single nucleotide variant disrupting protein localization in neuronal dendritic spines. Clin Genet. 2018;94(6):581–5. 30238967 10.1111/cge.13450

[CR80] Tatavarty V, Sun Q, Turrigiano GG. How to scale down postsynaptic strength. J Neurosci. 2013;33(32):13179–89. 23926271 10.1523/JNEUROSCI.1676-13.2013 PMC3735890

[CR81] Trepanier CH, Jackson MF, MacDonald JF. Regulation of NMDA receptors by the tyrosine kinase Fyn. FEBS J. 2012;279(1):12–9. 21985328 10.1111/j.1742-4658.2011.08391.x

[CR82] Viana VT, Durcan L, Bonfa E, Elkon KB. Ribosomal P antibody: 30 years on the road. Lupus. 2017;26(5):453–62. 28394227 10.1177/0961203317690243

[CR83] Williamson SL, Ellaway CJ, Peters GB, Pelka GJ, Tam PP, Christodoulou J. Deletion of protein tyrosine phosphatase, non-receptor type 4 (PTPN4) in twins with a Rett syndrome-like phenotype. Eur J Hum Genet. 2015;23(9):1171–5. 25424712 10.1038/ejhg.2014.249 PMC4538211

[CR84] Won S, Roche KW. Regulation of glutamate receptors by striatal-enriched tyrosine phosphatase 61 (STEP(61) ). J Physiol. 2021;599(2):443–51. 32170729 10.1113/JP278703 PMC11526339

[CR85] Won S, Levy JM, Nicoll RA, Roche KW. MAGUKs: multifaceted synaptic organizers. Curr Opin Neurobiol. 2017;43:94–101. 28236779 10.1016/j.conb.2017.01.006 PMC5447471

[CR86] Won S, Incontro S, Li Y, Nicoll RA, Roche KW. The STEP(61) interactome reveals subunit-specific AMPA receptor binding and synaptic regulation. Proc Natl Acad Sci U S A. 2019;116(16):8028–37. 30936304 10.1073/pnas.1900878116 PMC6475416

[CR87] Xu F, Plummer MR, Len GW, Nakazawa T, Yamamoto T, Black IB, et al. Brain-derived neurotrophic factor rapidly increases NMDA receptor channel activity through Fyn-mediated phosphorylation. Brain Res. 2006;1121(1):22–34. 17045972 10.1016/j.brainres.2006.08.129

[CR88] Xu J, Kurup P, Foscue E, Lombroso PJ. Striatal-enriched protein tyrosine phosphatase regulates the PTPalpha/Fyn signaling pathway. J Neurochem. 2015;134(4):629–41. 25951993 10.1111/jnc.13160 PMC4516628

[CR89] Yang X, Gong R, Qin L, Bao Y, Fu Y, Gao S, et al. Trafficking of NMDA receptors is essential for hippocampal synaptic plasticity and memory consolidation. Cell Rep. 2022;40(7):111217. 35977502 10.1016/j.celrep.2022.111217

[CR90] Yong XLH, Zhang L, Yang L, Chen X, Tan JZA, Yu X, et al. Regulation of NMDA receptor trafficking and gating by activity-dependent CaMKIIalpha phosphorylation of the GluN2A subunit. Cell Rep. 2021;36(1):109338. 34233182 10.1016/j.celrep.2021.109338 PMC8313361

[CR91] Zameer S, Ali J, Vohora D, Najmi AK, Akhtar M. Development, optimisation and evaluation of chitosan nanoparticles of alendronate against Alzheimer’s disease in intracerebroventricular streptozotocin model for brain delivery. J Drug Target. 2021;29(2):199–216. 32876502 10.1080/1061186X.2020.1817041

[CR92] Zamzow DR, Elias V, Acosta VA, Escobedo E, Magnusson KR. Higher levels of phosphorylated Y1472 on GluN2B subunits in the frontal cortex of aged mice are associated with good spatial reference memory, but not cognitive flexibility. Age. 2016;38(3):50. 27094400 10.1007/s11357-016-9913-2 PMC5005925

[CR93] Zarfeshani A, Carroll KR, Volpe BT, Diamond B. Cognitive impairment in SLE: mechanisms and therapeutic approaches. Curr Rheumatol Rep. 2021;23(4):25. 33782842 10.1007/s11926-021-00992-1 PMC11207197

[CR94] Zhu J, Shang Y, Zhang M. Mechanistic basis of MAGUK-organized complexes in synaptic development and signalling. Nat Rev Neurosci. 2016;17(4):209–23. 26988743 10.1038/nrn.2016.18

