# Supplementary material for: Anti-P antibodies that impair memory perturb hippocampal glutamatergic receptor trafficking, synapse structure and microglia
Source: Mol Med. 2025 Sep 26;31:290. doi: 10.1186/s10020-025-01339-7 (PMC12465742; doi:10.1186/s10020-025-01339-7)
Supplement: Supplementary file 1 — Supplementary Material 1 [file 10020_2025_1339_MOESM1_ESM.pdf]

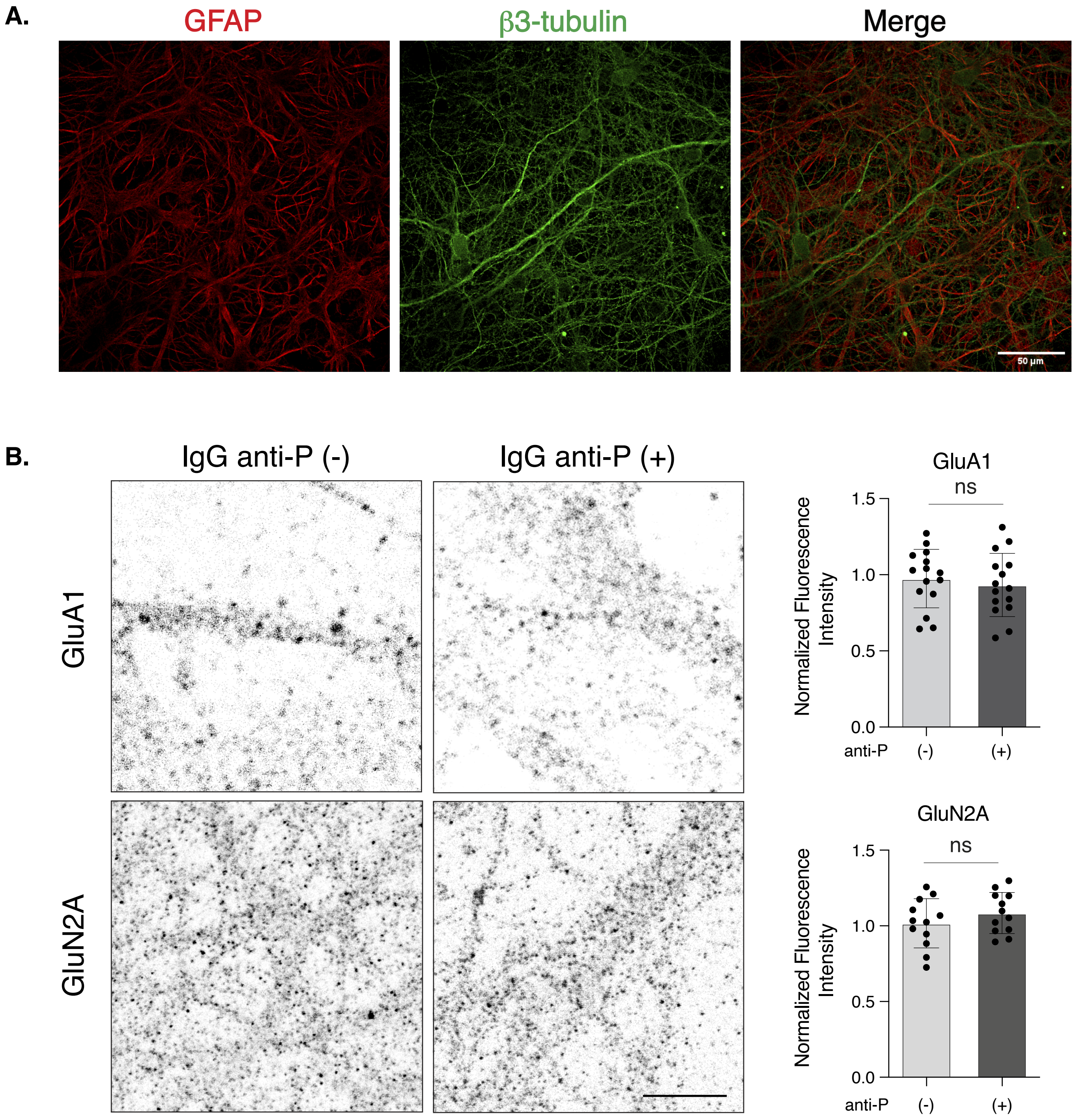

Supplementary Figure 1: Primary hippocampal culture and the effects of anti-P antibodies on total AMPA and NMDA receptor levels.

Hippocampal primary culture at 21DIV were fixed, permeabilized and immunostained for:
A. Astrocyte maker (GFAP-Red) and neuronal marker (β3-tubulin) to depict the complex/intrincate nature the preparation. B. NMDAR subunit GluN2A and AMPAR subunit GluA1 with or without treatment with anti-P antibodies for 1 hour. Total Fluorescence was quantified in 12 independent fields, no statistical differences were observed (Scale Bar 10 μm, n=12, P value =0.5772, Unpaired t-test).
