# Supplementary material for: Anti-P antibodies that impair memory perturb hippocampal glutamatergic receptor trafficking, synapse structure and microglia
Source: Mol Med. 2025 Sep 26;31:290. doi: 10.1186/s10020-025-01339-7 (PMC12465742; doi:10.1186/s10020-025-01339-7)
Supplement: Supplementary file 2 — Supplementary Material 2 [file 10020_2025_1339_MOESM2_ESM.pdf]

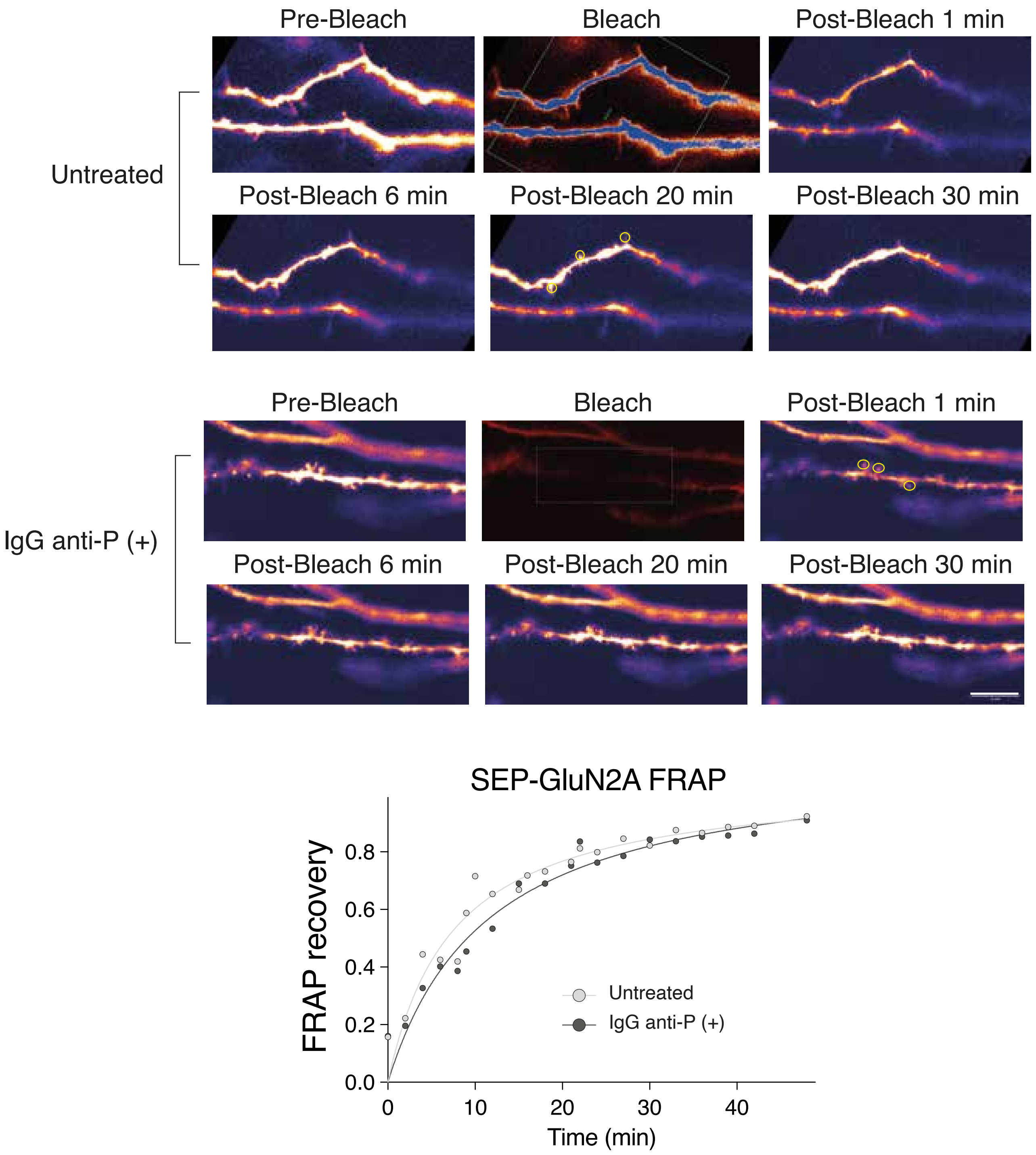

Supplementary Figure 2: Sep-GluN2A FRAP.
21 days hippocampal primary cultured neurons transfected with Sep-GluN2A were grown in live imaging 35 mm dishes. 50um long regions were photobleached and then fluorescence was recorded during 50 min. Pre and post bleaching representative images are shown in intensity pseudocolor LUT. Bleached ROI is shown in the second picture. Individual ROIs spanning individual dendritic spines are shown (Bar: 10 μm). Quantification of individual spines fluorescence recovery shows a similar pattern reaching a plateau after 20 minutes. A small difference between anti-P(+) IgG and untreated neurons can be observed within the first 5 minutes (n=1).
