# Supplementary material for: Anti-P antibodies that impair memory perturb hippocampal glutamatergic receptor trafficking, synapse structure and microglia
Source: Mol Med. 2025 Sep 26;31:290. doi: 10.1186/s10020-025-01339-7 (PMC12465742; doi:10.1186/s10020-025-01339-7)
Supplement: Supplementary file 3 — Supplementary Material 3 [file 10020_2025_1339_MOESM3_ESM.pdf]

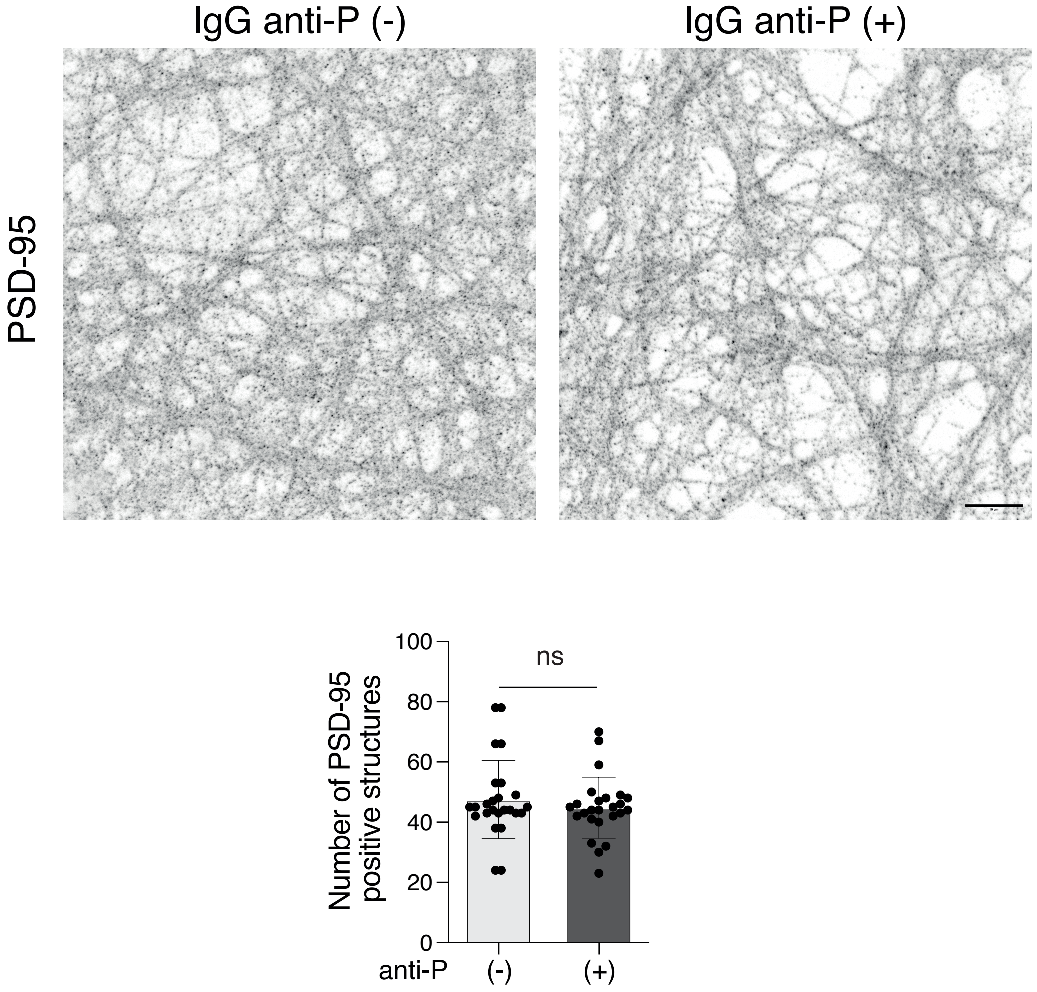


**Supplementary Figure 3: The effects of anti-P antibodies on total PSD-95 protein levels in primary hippocampal culture.**

Primary hippocampal neurons cultured in 12mm coverslips were treated for 1hr with 25μg/ml of anti-P(+) serum or preimmune serum (anti-P(-)), then washed, fixed and stained with rabbit anti-PSD95 antibody. Images were acquired in a Leica TCS SP8 confocal microscope and 25μmx20μm ROIs were drawn along dendrites. Particle analyzer plugin (ImageJ) was used to quantify the number of PSD-95 structures. Representative images are shown (Scale Bar =10 μm, n = 25 ROIs from two independent experiments, unpaired t-test).
