# Supplementary material for: Anti-P antibodies that impair memory perturb hippocampal glutamatergic receptor trafficking, synapse structure and microglia
Source: Mol Med. 2025 Sep 26;31:290. doi: 10.1186/s10020-025-01339-7 (PMC12465742; doi:10.1186/s10020-025-01339-7)
Supplement: Supplementary file 4 — Supplementary Material 4 [file 10020_2025_1339_MOESM4_ESM.pdf]

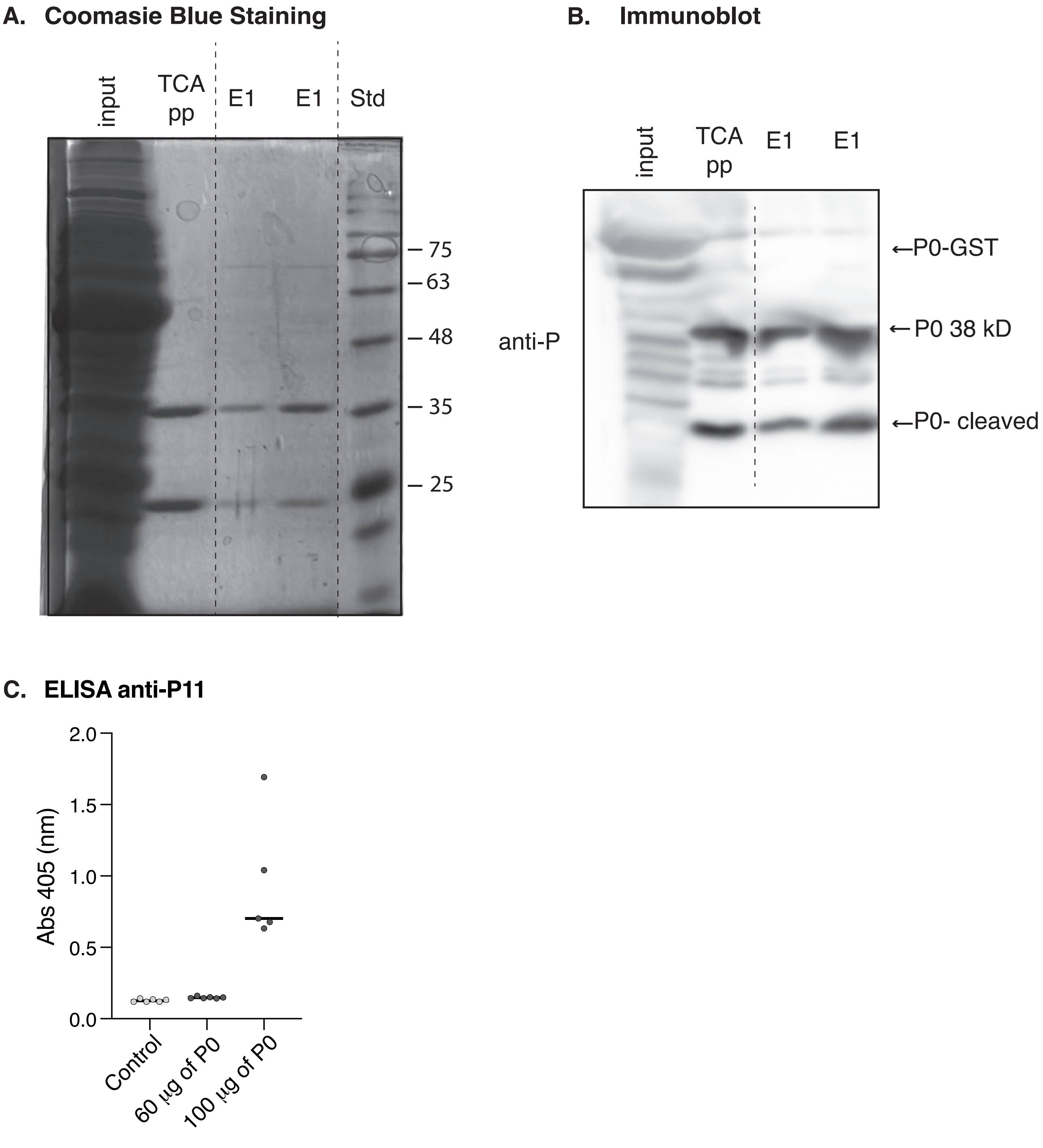

Supplementary Figure 3: Recombinant protein purification of P0-GST and ELISA analysis against the syntetic P11-epitope peptide.

E. coli strain DH5α expressing recombinant P0 protein fused to GST in pGEX-4T-3 vector were used for expression of recombinant proteins. The bacteria lysates were added to a Glutathione-Superflow Resin column, to cut and separate P0 from GST Thrombin protease was used. The resulting fractions, proteins precipitated with TCA (TCA pp), first and second elution from column (E1 and E2) were analyzed by SDS-PAGE and stained with Coomassie in A. or transferred to PVDF membranes to immunoblot using anti-P antibody in B. C57BL/6 female mice immunized with 60 or 100 μg of recombinant P0 protein as indicated in C. No anti-P antibody titers were detected 5 weeks after immunization when 60 μg were used.
