# Supplementary material for: Anti-P antibodies that impair memory perturb hippocampal glutamatergic receptor trafficking, synapse structure and microglia
Source: Mol Med. 2025 Sep 26;31:290. doi: 10.1186/s10020-025-01339-7 (PMC12465742; doi:10.1186/s10020-025-01339-7)
Supplement: Supplementary file 5 — Supplementary Material 5 [file 10020_2025_1339_MOESM5_ESM.pdf]

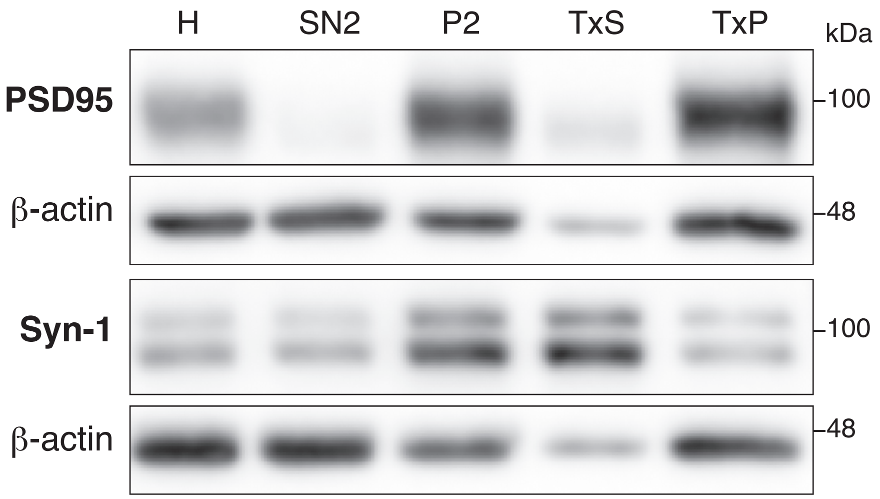

Supplementary Figure 4: Characterization of postsynaptic densities (PSD) and total membranes fractions (P2). PSD protein enrichment from hippocampus of C57BL6 mice was verified by immunoblot with the presynaptic marker Synapsin-1 (Syn-1) and postsynaptic marker PSD-95. Hippocampal crude extract (H), cytosolic supernatant and microsomal fraction (SN2), total membrane fraction (P2), presynaptic and total membranes without PSD (TxS) and postsynaptic densities (TxP).
